# The efficient synthesis of three-membered rings *via* photo- and electrochemical strategies

**DOI:** 10.1039/d4sc02512a

**Published:** 2024-08-14

**Authors:** Xinyu Han, Na Zhang, Qiannan Li, Yu Zhang, Shoubhik Das

**Affiliations:** a Shanghai Frontiers Science Center for Chinese Medicine Chemical Biology, Institute of Interdisciplinary Integrative Medicine Research, Shanghai University of Traditional Chinese Medicine No. 1200, Cailun Road Shanghai 201203 China yzhang@shutcm.edu.cn; b Department of Laboratory Medicine, Shanghai East Hospital, Tongji University School of Medicine Shanghai China; c Department of Chemistry, University of Bayreuth Bayreuth 95447 Germany Shoubhik.Das@uni-bayreuth.de; d School of Chemistry and Chemical Engineering, Henan Normal University Xinxiang Henan 453007 People's Republic of China

## Abstract

Three-membered rings, such as epoxides, aziridines, oxaziridines, cyclopropenes, vinyloxaziridines, and azirines, are recognized as crucial pharmacophores and building blocks in organic chemistry and drug discovery. Despite the significant advances in the synthesis of these rings through photo/electrochemical methods over the past decade, there has currently been no focused discussion and updated overviews on this topic. Therefore, we presented this review article on the efficient synthesis of three-membered rings using photo- and electrochemical strategies, covering the literature since 2015. In this study, a conceptual overview and detailed discussions were provided to illustrate the advancement of this field. Moreover, a brief discussion outlines the current challenges and opportunities in synthesizing the three-membered rings using these strategies.

## Abstract 2

Three-membered rings, such as epoxides, aziridines, oxaziridines, cyclopropenes, vinyloxaziridines, and azirines, are recognized as crucial pharmacophores and building blocks in organic chemistry and drug discovery.

## Graphical abstract

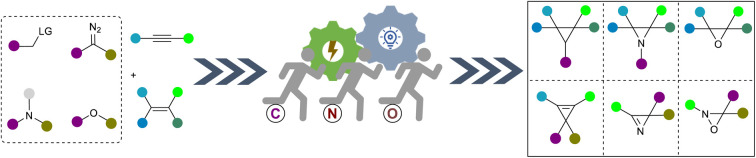

## 1 Introduction

**Fig. 1:**
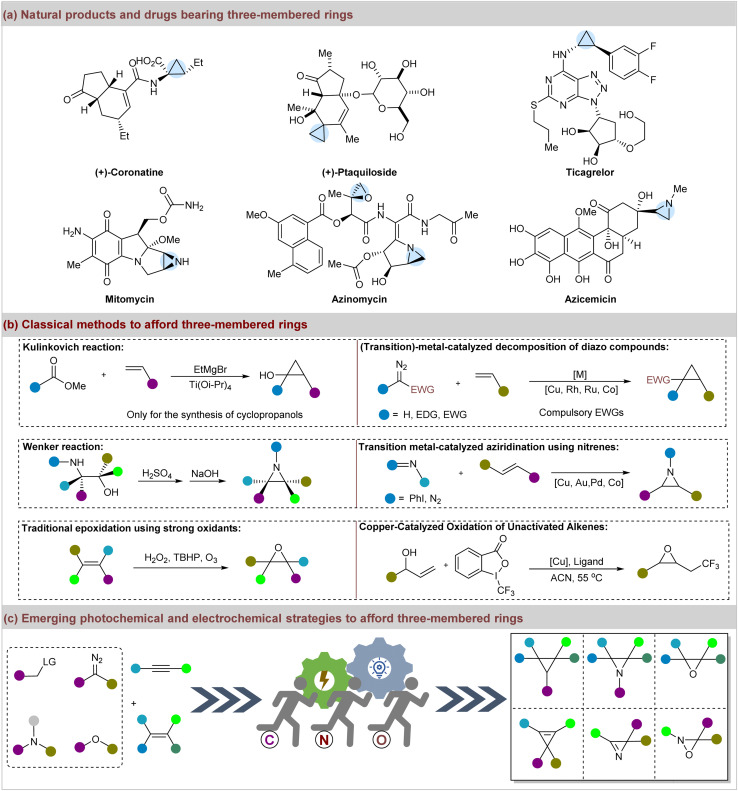
(a) Representative drugs and natural products bearing three-membered rings; (b) classical methods to afford cyclopropanes, aziridines, and epoxides. (c) Emerging visible-light mediated and electrochemical strategies to afford three-membered rings.

Three-membered rings, including cyclopropanes, epoxides, aziridines, oxaziridines, cyclopropenes, vinyloxaziridines, and azirines, play crucial roles as pharmacophores in medicinal chemistry, and are prevalent in natural products and drugs.^1–5^ They are also commonly present in pesticides, perfumes, and specialty chemicals.^6–9^ Additionally, these motifs are frequently identified in the top 200 highest-grossing pharmaceutical products and other approved drugs.^10^ Notably, cyclopropyl drugs such as the phytotoxin coronatine, anticancer agent ptaquiloside, and antiplatelet agent ticagrelor are well-known examples.^11–14^ Furthermore, diverse natural products with three-membered rings exhibit significant bioactive properties.^15,16^ For instance, mitomycin and azinomycin are noted for their antibiotic and antitumor activities, and azicemicin is effective against Gram-negative bacteria and mycobacteria (Fig. 1a).^17–19^ In addition to their prevalence in pharmaceuticals and natural products, three-membered rings are utilized as foundational components in synthetic chemistry.^20–24^ Moreover, achieving sustainable synthesis of structurally diverse three-membered rings has long been a goal in organic and medicinal chemistry, with numerous classic methods reported for their preparation (Fig. 1b).^25–27^ Initially, it was discovered that nature constructs cyclopropanes through biosynthetic pathways involving the ring closure of alkenes and carbocations, which inspired synthetic chemists to mimic these processes.^28^ Noteworthy synthetic routes include the Freund reaction, Kishner cyclopropane synthesis, the Kulinkovich reaction, and the Simmons–Smith reaction.^29–33^ In addition, intramolecular methods for constructing aziridines, such as the Wenker-type reaction of amino alcohols or their derivatives, have been reported.^34–36^ Catalytic alkene aziridination is another efficient method for the synthesis of aziridines.^37^ Nowadays, the established strategies for achieving aziridination have included the application of nitrenes, nitrogen-centered radicals, halonium ions, and oxaziridines.^38–41^ The epoxidation of alkenes typically necessitates strong oxidants such as hydrogen peroxide (H_2_O_2_), *tert*-butyl hydroperoxide (TBHP), hypervalent iodine, and ozone, or classic reactions including the Darzens condensation and Corey–Chaykovsky reaction.^42–45^ Transition-metal catalysis has been another prevalent method to afford epoxides efficiently.^46,47^ Furthermore, the synthesis of other three-membered rings, such as cyclopropenes, vinyloxaziridines, and azirines, has also been well-established.^48–58^

Despite the advancements in obtaining three-membered rings, numerous strategies still involve high temperatures, external oxidants, and poor atom economy.^59,60^ In this regard, the visible-light-mediated and electrocatalytic syntheses of three-membered frameworks have flourished^61,62^ and have attracted growing interest as environmentally friendly and potent approaches (Fig. 1c).^63,64^ In fact, significant progress has been made in the construction of three-membered rings using these innovative approaches. However, there is a lack of focus on this topic.^65–67^ To address this gap, we aimed to provide a comprehensive and critical review of recent advancements in the synthesis of three-membered rings *via* visible-light-mediated and electrochemical strategies since 2015. By focusing on homogeneous systems, we anticipate that this review will be valuable to readers of organic synthesis, catalysis, and medicinal chemistry.

## 2 Conceptual overview of the synthesis of three-membered rings *via* photo/electrochemical strategies

**Fig. 2:**
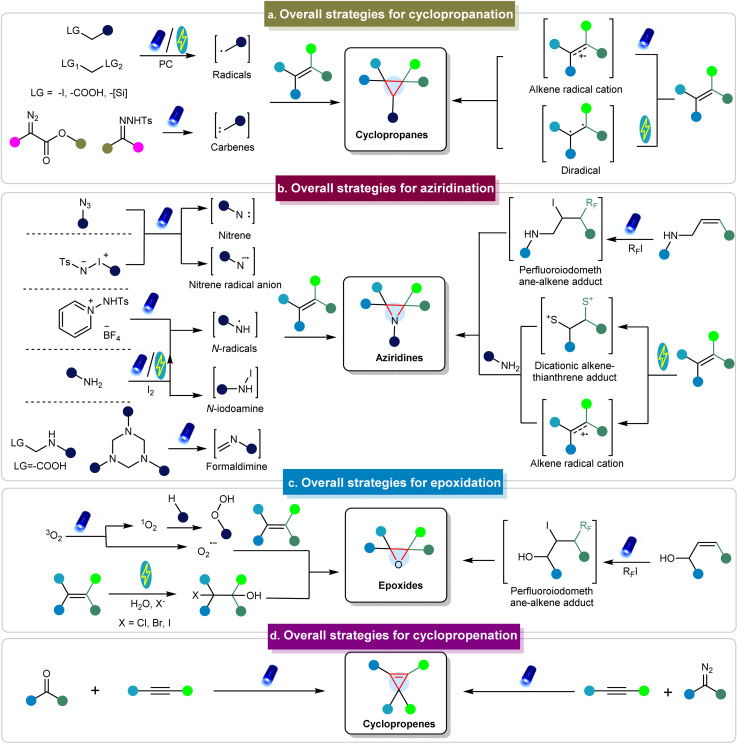
Conceptual overview of synthesizing three-membered rings *via* photo/electrochemical approaches.

To illustrate the formation of three-membered ring compounds *via* photo/electrochemical methods, Fig. 2 illustrates an overview of key mechanistic principles. There are generally two types of initiation pathways for the construction of three-membered ring compounds, including the donor-initiated and acceptor-initiated methods. The donor-initiated methods for synthesizing cyclopropanes typically involve the generation of carbon radicals or carbenes. Carbon radicals are generally derived from radical precursors containing leaving groups (such as halogens, carboxylic acids, and silicon reagents)^68–72^ that depart in the presence of catalysts, light, or electricity to form radicals. These radicals can further react with alkenes to produce cyclopropanes. On the other hand, carbenes are often produced from diazo esters^73^ and other diazo precursors such as *N*-tosylhydrazones to achieve cyclopropanation.^74,75^ In contrast, acceptor-initiated methods can activate alkenes through the formation of radical cations or diradicals under photo/electrocatalytic conditions to facilitate cyclopropanation.^76–78^

In terms of donor initiation methods for synthesizing aziridine compounds, several pathways involve the formation of nitrenes, nitrene radical anions, *N*-radicals, *N*-iodoamine, and formaldimine intermediates derived from various precursors, facilitated by photo/electrocatalysis (Fig. 2).^79–83^ These intermediates can subsequently react with alkenes to produce the corresponding aziridines. Acceptor-initiated methods involve the formation of alkene radical cations and dicationic alkene–thianthrene adducts.^84^ Moreover, alkenes can react with perfluoroalkyl iodides under photocatalytic conditions to generate perfluoroiodomethane–alkene adducts, which then react with amines to form aziridines.^85^ Epoxides are typically prepared *via* photochemical pathways involving O_2_, including the pathways using singlet oxygen (^1^O_2_) or superoxide radical anions (O_2_˙^−^).^86^ In addition, epoxides can be generated *via* perfluoroiodomethane–alkene adducts.^87^ The electrochemical synthesis of epoxides typically involves H_2_O and requires the halogen atom as the leaving group.^88–90^ Cyclopropenes are primarily synthesized through reactions between carbenes and alkynes.^91,92^ In addition to these methods, other mechanisms have also been employed to obtain three-membered rings.^93–96^ However, there are limited examples of the synthesis of other three-membered rings *via* photo/electrochemical methods, as detailed in the following sections. These methods are discussed in detail in the subsequent sections.

## 3 Synthesis of cyclopropanes by photo/electrocatalysis

As mentioned earlier, cyclopropanes are crucial structural elements in drug molecules. Various methods have been developed for the synthesis of cyclopropanes using diverse catalytic systems and specially designed substrates. Under mild photo/electrocatalytic reaction conditions, radical precursors can be derived from dichloromethanes, halogenated methyl silicates, or carboxylic acids;^68–70^ and diazo compounds,^73^ trifluoroacetyl silanes,^74^ and *N*-tosylhydrazones^75^ serve as the carbene sources. In addition, alkenes can be directly activated to form alkene radical cations or diradicals, leading to cyclopropane products.^76–78^ These advancements have significantly enhanced the efficient construction of structurally diverse cyclopropanes.

### 3.1 Cyclopropanation through the construction of radical intermediates

The generation of radical intermediates to produce cyclopropanes has been a pivotal strategy over the past decade. In 2002, the Nédélec group introduced an electrocatalytic method for cyclopropanation, involving the reaction between reactive alkenes and activated 1,1,1-trichloro compounds.^97^ This method allowed the generation of a variety of chlorine-substituted cyclopropanes in acceptable yields (25–76%). Although the substrate scope of this reaction was limited with no entirely elucidated mechanism, it can be notable for its significant advantage of not requiring hazardous reagents such as diazo compounds or diazirines. Navarro *et al.* further investigated the mechanism through cyclic voltammetry and electrolysis in 2011.^98^ They discovered that cyclopropanation occurred *via* radical or anionic intermediates, and that subsequent iron catalysis facilitated the coupling reactions by preventing dimer formation in radical-mediated pathways. Conversely, in the anionic pathway, the electrocatalytic reduction of dimethyl itaconate to the corresponding carbanion, followed by nucleophilic addition, led to the formation of cyclopropane products.

**Scheme 1:**
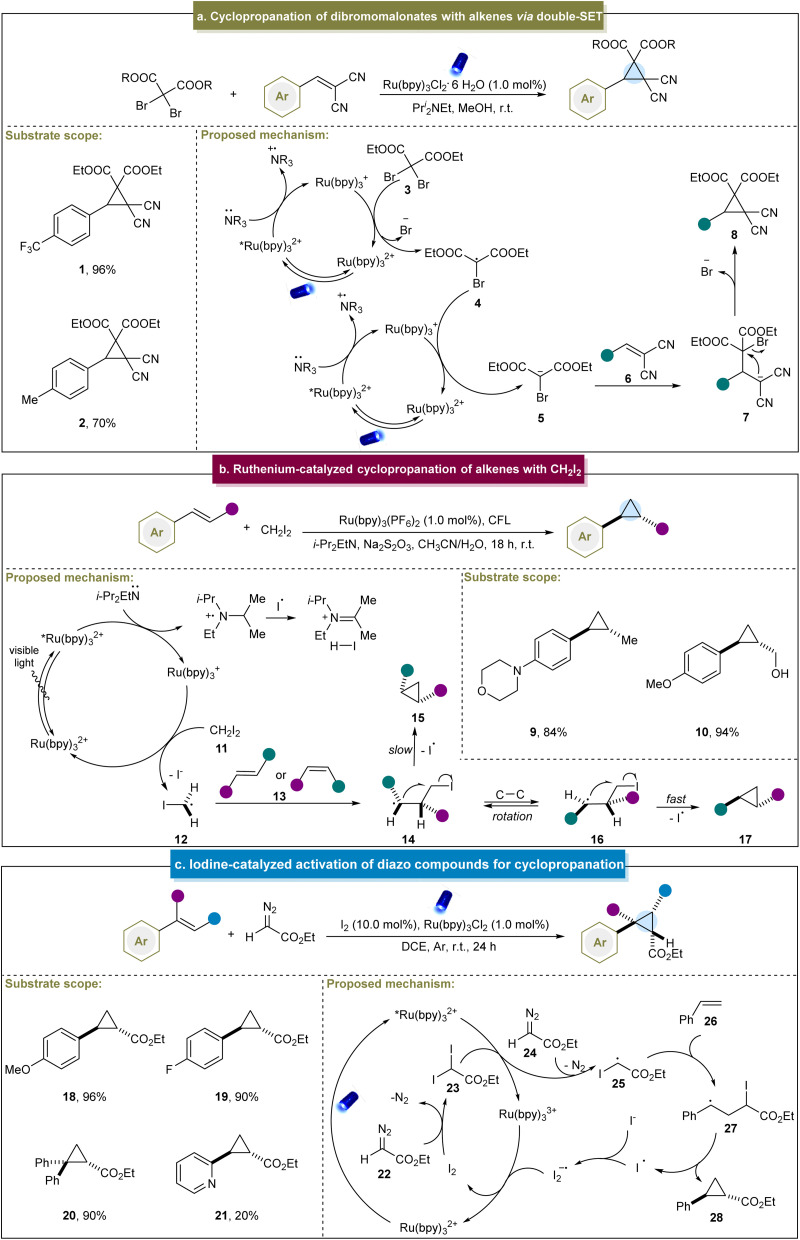
(a) Cyclopropanation of dibromomalonates with alkenes *via* double-SET. (b) Ruthenium-catalyzed cyclopropanation of alkenes. (c) Iodine-catalyzed activation of diazo compounds for cyclopropanation.

In 2015, Guo *et al.* pioneered a visible light-induced strategy for the cyclopropanation of dibromomalonates with 2-benzylidenemalononitrile. This method involved generating a carbanion through a double single-electron transfer (SET) mechanism (Scheme 1a).^99^ This strategy was feasible under sunlight in open air, highlighting its sustainability and ease of application. Initially, the Ru(bpy)_3_^2+^ photocatalyst absorbed photons under visible-light irradiation, entering an excited state. Subsequently, this excited species captured an electron from the amine *via* a SET process, resulting in the formation of highly reduced Ru(bpy)_3_^+^. Subsequently, dibromomalonate (3) underwent the first electron transfer process, lost a bromide ion to generate a dibromomalonate radical (4) and regenerated the original photocatalyst. Subsequent catalytic cycles involved a second electron transfer process that converted the carbon-centered radical into the desired dibromomalonate anion (5). Michael addition occurred to an electron-deficient alkene (6), followed by intramolecular nucleophilic substitution, ultimately yielding cyclopropane (8) as the final product. Although this was a breakthrough to access cyclopropanes under photocatalytic conditions, this method was restricted to specific alkenes bearing electron-withdrawing groups, and thereby led to poor substrate scope.

Similarly, the group of Suero reported the stereoselective cyclopropanation of styrenes *via* photoredox catalysis.^68^ This method facilitated the selective formation of *trans*-cyclopropanes using both *E*- and *Z*-type anetholes and CH_2_I_2_. In particular, high yields were obtained from aromatic alkenes with unprotected groups such as hydroxyl (10). In their reaction, the photoexcited state *[Ru(bpy)_3_]^2+^ underwent the SET process with *N,N*-diisopropylethylamine to generate [Ru(bpy)_3_]^+^ (Scheme 1b). Subsequently, [Ru(bpy)_3_]^+^ donated an electron to CH_2_I_2_ (11) to produce an iodomethyl radical (12) and in the presence of *E*, *Z*-alkenes, was converted into intermediates (14) and (15). Finally, the cyclization step facilitated the homolytic substitution of intermediate (16), favoring a *trans* configuration of the R^1^ and R^2^ groups. This method enabled the selective formation of *trans*-cyclopropanes under mild conditions and effectively controlled the stereochemistry of the *E*- or *Z*-type aromatic alkenes. However, several challenges remain; for example, aromatic alkenes with moderate/strong electron-withdrawing groups exhibited lower yields. On this basis, Suero *et al.* extended their research by utilizing diiodomethane in the photocatalytic cyclopropanation of α,β-unsaturated carbonyl compounds.^100^

In 2018, the group of Li reported a photoinduced iodine-catalyzed diazo activation method for the synthesis of cyclopropanes.^101^ This approach proved effective for the arene-substituted alkenes bearing both electron-donating and electron-withdrawing groups, yielding the desired products in good to excellent yields (18–20). However, the yield decreased significantly for aromatic alkenes containing heteroatoms (21). Mechanistically, diazo compound (22) initially reacted with I_2_ to form diiodo compound (23), which converted *[Ru(bpy)_3_]^2+^ into [Ru(bpy)_3_]^3+^ (Scheme 1c). Subsequently, an active intermediate was formed when the diiodo compound reacted with 24, generating a carbon radical (25) through the SET process. This carbon radical (25) attacked the alkene to form another radical intermediate (27). Ultimately, cyclopropane was produced through the release of an iodine radical. However, it was challenging for aliphatic and other unactivated alkenes to undergo cyclopropanation through this strategy and diazo compounds are only applicable to diazo esters.

Based on the pioneering studies mentioned above, Ooi *et al.* utilized halogen activation to generate radical species, achieving boryl cyclopropanation with α-MIDA-boryl styrenes and diiodoborylmethane (MIDA: *N*-methyliminodiacetyl) under visible-light irradiation.^102^ This approach is similar to the previous halogen activation pathways but exhibited limited substrate compatibility and lacked detailed mechanistic insights, owing to insufficient experimental investigations. Furthermore, Suero *et al.* developed a photocatalyst-free method employing *gem*-diiodomethyl carbonyl reagents to access a variety of cyclopropanes.^103^ This protocol demonstrated the efficacy for various substituted styrenes. Moreover, the late-stage functionalization of bioactive molecule derivatives highlighted the synthetic versatility of this method. Unfortunately, styrenes containing electron-withdrawing groups (such as –CF_3_) and vinylpyridines were not suitable for this reaction. Tokuyama *et al.* (2021) developed an intramolecular cyclopropanation strategy using α-bromo-β-keto esters and alkenes.^104^ Under photocatalytic conditions, α-bromo-β-keto esters underwent bromide ion loss to generate a carbon radical intermediate. This intermediate participated in the intramolecular radical addition to the alkene, followed by cyclization to produce cyclopropane products. Additionally, Maji *et al.* introduced a concise method for the rapid and efficient synthesis of various monosubstituted 1,1- and 1,2-disubstituted cyclopropanes from unactivated 1,3-alkyl electrophiles.^105^ This mild approach exhibited tolerance towards diverse substrates that were previously ineffective, such as alkynes, free hydroxyls, and terminal alkenes. Inspired by this work, Liu *et al.* recently described photoredox-catalyzed boron cyclopropanation of diverse alkenes, including styrenes and aliphatic alkenes.^106^

**Scheme 2:**
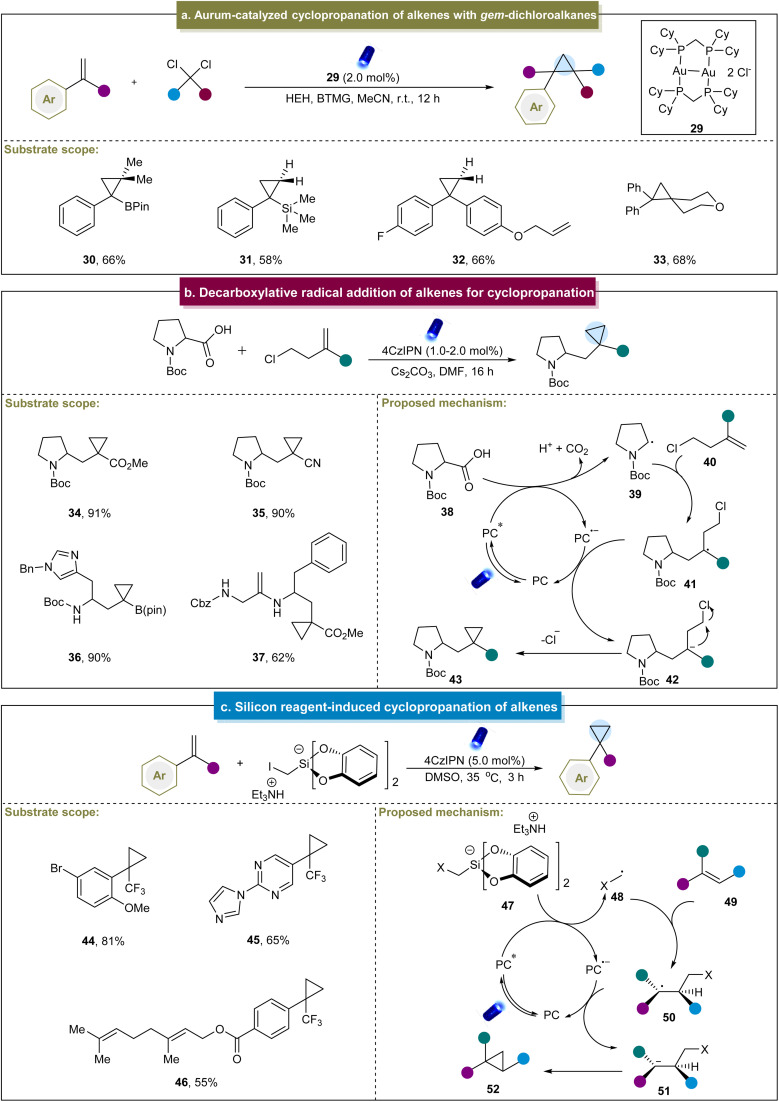
(a) Aurum-catalyzed cyclopropanation of alkenes with *gem*-dichloroalkanes. (b) Decarboxylative radical addition of alkenes for cyclopropanation. (c) Silicon reagent-induced cyclopropanation of alkenes.

Later, the group of Carreira carried out intermolecular cyclopropanation using α-bromo-β-ketoesters and α-bromo-maleic acid esters with unactivated alkenes in the presence of an organic photocatalyst.^107^ This reaction demonstrated broad functional group tolerance and excellent compatibility with mono-, di- and tri-substituted alkenes, yielding highly substituted cyclopropane compounds. Mechanistic studies indicated that the reaction proceeded primarily through catalyst reduction by α-bromo-β-ketoesters, generating a carbon-centered radical that engaged in subsequent reactions with the alkene to afford cyclopropane products. Recently, Xie and co-workers reported the gold-catalyzed cyclopropanation between styrenes and *gem*-dichloroalkanes (Scheme 2a).^108^ In this work, diverse *gem*-dichloroalkanes were used as chloroalkyl radical equivalent precursors for cyclopropanation with 1,1-disubstituted styrenes. Unfortunately, this method was not feasible for aliphatic alkenes, and thereby led to poor substrate scope. The mechanism of this reaction primarily involved the interaction of a photocatalyst and Hantzsch ester (HEH), forming a potentially weakly bound gold complex. This complex absorbs visible light to generate an excited-state species that coordinated with dichloromethane to produce an exciplex, thereby releasing chloromethyl radicals. These radicals could be rapidly introduced to the alkenes, facilitating the formation of cyclopropane products through radical 3-*exo-tet* cyclization. Additionally, Charette *et al.* developed a visible-light-mediated borosilylcyclopropanation method for styrene derivatives.^109^ Mechanistic studies have revealed that the cyclopropanation process can be stabilized by a four-membered-ring boronate anion. The transient radical anion decomposes into an iodo-boromethylsilane radical, which subsequently undergoes a series of reactions to produce cyclopropane. Furthermore, Vassilikogiannakis *et al.* employed dihaloalkanes as cyclopropanation reagents to achieve metal-free photocatalytic cyclopropanation through the XAT mechanism.^110^ Notably, complicated colibactin metabolite compounds were also accommodated in this system to furnish valuable natural product derivatives.

In addition to the radical formation *via* halogen elimination, Aggarwal *et al.* devised an elegant method for the synthesis of cyclopropanes. This method utilized carboxylic acids and chloroalkyl alkene substrates through a decarboxylative radical addition-polar cyclization cascade.^69^ The strategy was suitable for various aliphatic alkenes containing different electron-withdrawing groups and carboxylic acids with multiple functional groups. Initially, carboxylic acid (38) underwent the SET with an excited-state catalyst (Scheme 2b). This process led to the deprotonation of the carboxylic acid and liberation of CO_2_, generating a carbonyl radical (39). The radical attacked the homoallyl chloride (40), forming a stable alkyl radical (41), followed by the second SET with PC˙^−^. Finally, the resulting stable carbon anion (42) underwent polar 3-*exo-tet* cyclization to yield the cyclopropane product. Similarly, Sureshkumar *et al.* reported a method for the synthesis of 2-acyclic cyclopropane derivatives by coupling α-keto acids with cyclopropenes.^111^ The key step involved SET-mediated decarboxylation to generate the corresponding acyl radical, which was selectively added to one side of cyclopropene to form cyclopropanes. This study advanced the synthetic versatility of cyclopropenes, whereas the mechanistic details were already understood.

In addition to the aforementioned methods, Molander *et al.* pioneered the development of a novel bifunctional reagent, triethylammonium bis(catecholato)iodomethylsilicate, for cyclopropanation in 2018.^70^ This method facilitated the straightforward synthesis of cyclopropanes from α-trifluoromethyl styrenes, yielding moderate to excellent yields (44 and 45). Additionally, in substrates with multiple olefinic double bonds, cyclopropanation selectively occurred on the electron-deficient trifluoromethyl alkenes (46). A plausible mechanism was proposed (Scheme 2c), beginning with the formation of the excited state of 4CzIPN under visible light irradiation. Subsequent reductive quenching of 4CzIPN* by halomethyl silicate 47 generated the halomethyl radical 48, which underwent a Giese-type addition to form adduct 50. Further SET reduction of 50 by the reduced 4CzIPN produced anion 51, regenerating 4CzIPN to its ground state. Finally, anion 51 underwent 3-*exo-tet* cyclization to yield cyclopropane 52. Although this method was limited to specific α-trifluoromethyl styrenes, it provided a novel approach for the subsequent cyclopropane's construction efforts.

Building on this refined method, several subsequent studies have utilized similar bifunctional reagents. Ouyang *et al.* developed a comparable strategy for cyclopropanation using chloromethyl silicates as the methyl source.^112^ In addition to the α-substituted vinyl phosphonates, a range of Michael acceptors have been shown to be suitable substrates for photocatalytic cyclopropanation. Notably, vinyl phosphonate, a complex derivative of estrone, exhibited high reactivity, yielding the desired cyclopropane product. Continuing with this approach, the Molander group extended the application of this reagent, outlining a cyclization process to synthesize 1,1-disubstituted cyclopropanes from homoallylic tosylates.^113^ In this approach, cyclopropanes were generated by the reaction of alkyl radicals with alkenes, where homoallylic tosylate served as the leaving group. The method exhibited high tolerance for structural diversity in nucleophilic reagents and enabled the rapid and efficient synthesis of a broad array of cyclopropane products without requiring strongly polarized alkenes. However, it was limited to styrenes containing electron-withdrawing groups. Chen *et al.* reported the photoredox-catalyzed cyclopropanation of 1,1-disubstituted alkenes using halogens as the leaving groups.^114^ This reaction involved the alkyl and acyl radicals in a radical addition-anionic cyclization process, utilizing the bis-catecholato silicates as radical precursors.

In 2020, Zhang *et al.* introduced a protocol for the visible-light-induced cyclopropanation of alkenes using bromomethyl silicate as a methylene transfer reagent.^115^ This method enabled the cyclopropanation of terminal alkenes and demonstrated the feasibility of methyl transfer reactions to internal alkenes and inactivated 1,1-dialkyl ethylenes. Moreover, the synthesis of LG100268, a potent retinoid X receptor (RXR) agonist, validated the utility of this methylene transfer reaction. Subsequently, Du *et al.* reported the efficient cyclopropanation of alkenyl *N*-methylimino diacetyl boronates.^116^ Various MIDA cyclopropyl boronate derivatives were synthesized *via* photoredox-catalyzed cyclopropanation using chloro- or bromo-methyl silicate as the methylene source. Ohmiya *et al.* developed a method for the cyclopropanation of reactive alkenes by direct photoexcitation of borate under visible-light irradiation to generate iodomethyl radicals.^117^ This method offered a straightforward protocol for the cyclopropanation of dehydroamino acids (DHAAs), yielding the pharmacologically valuable cyclopropanyl amino acids. Recently, You *et al.* reported the efficient synthesis of methylene-unsubstituted cyclopropane-fused indolines *via* cyclopropanation between the indoles and iodomethylsilicates.^118^ However, the reaction was limited to *N*-protected and electron-deficient indoles, thus limiting its applicability. In 2022, Fang *et al.* reported the cyclopropanation of 1-alkyl-substituted ethenylphosphonates,^119^ which resulted in a series of alkyl phosphonate-substituted cyclopropane products. Fang *et al.* described a method for *N*-vinylimine cyclopropanation *via* photoredox catalysis.^120^ This cyclopropanation exhibited stereochemical control, and its synthetic utility was demonstrated through the deprotection of the amine protecting groups.

**Scheme 3:**
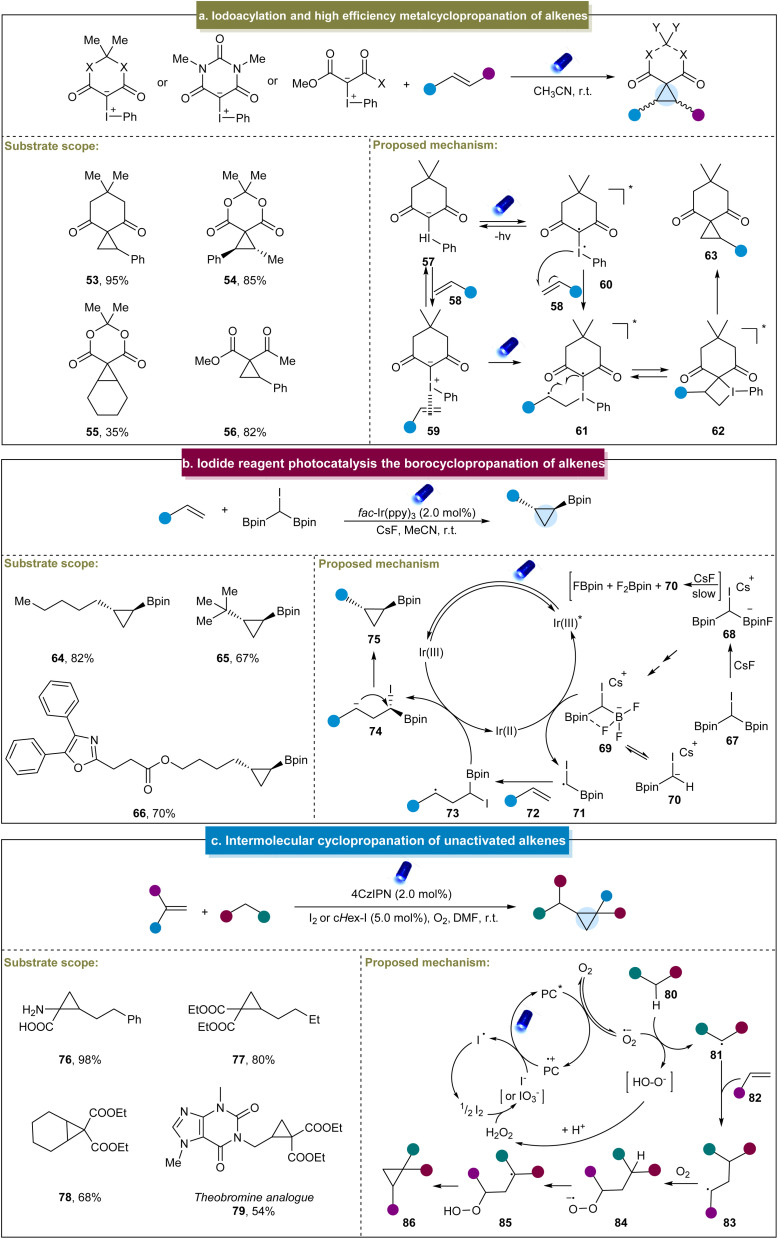
(a) Iodoacylation and high efficiency metalcyclopropanation of alkenes. (b) Iodide reagent photocatalysis and the borocyclopropanation of alkenes. (c) Intermolecular cyclopropanation of unactivated alkenes.

In addition to the bifunctional reagents developed by Molander *et al.*,^70^ in 2019, Murphy *et al.* achieved the synthesis of cyclopropanes by the direct irradiation of a mixture of iodonium ylides and aromatic alkenes using blue LEDs.^71^ This method efficiently yielded cyclopropanes from a variety of alkenes and iodonium ylides, with isolated yields ranging from excellent to good (53–55). Additionally, previously challenging aliphatic alkene substrates with medium reactivity were accommodated in this system (56). A one-pot method was developed using activated methylene ylide precursors. Furthermore, a plausible reaction mechanism was proposed based on the computational and experimental studies (Scheme 3a). The photoexcitation of iodonium ylide (57) induced the reversible formation of biradical intermediate (60), which then reacted with the alkene to form biradical (61). Cyclization resulted in the formation of iodocyclobutane (62), and subsequent reductive elimination of iodobenzene furnished the final cyclopropane product (63). Another plausible pathway involved the ground state of iodonium ylide, featuring an entirely positive charge on the iodine atom. This reaction was initiated by the alkene acting as a Lewis base towards the iodonium, thereby forming complex 59, which served as a precursor to form the biradical (61) under light irradiation. The subsequent cyclization and reductive elimination gave the final product.

Marder *et al.* developed a (bisborylmethyl)iodine reagent (CHI(Bpin)_2_) for the photoinduced cyclopropanation with aliphatic alkenes, yielding a range of 1,2-substituted cyclopropylboronates.^121^ This α-haloboronic ester easily synthesized from commercially available starting materials produced diverse cyclopropylboronates with high yields and excellent diastereoselectivity and demonstrated good tolerance towards various functional groups (64–66). However, this strategy was only applicable to terminal alkenes. The proposed mechanism for the reaction was deduced from the experimental data (Scheme 3b). Initially, the reaction of 67 with CsF produced a mixture of species, including trifluoroborate complex 69. Subsequently, the excited-state Ir(iii)* catalyst was reductively quenched by 69, generating the carbon-centered radical CHI(Bpin) 71. The alkyl radical was then added to the alkene to form radical intermediate (73), followed by the electron transfer from Ir(ii) to generate carbon anions (74) and restore the ground-state Ir(iii) photocatalyst. Finally, polar 3-*exo* cyclization occurred, resulting in the formation of the borylcyclopropane product.

More recently, Giri *et al.* discovered a photoinduced cyclopropanation between EWG-containing aliphatic alkenes and reactive methylene compounds.^72^ Under the standard reaction conditions, the process efficiently constructed cyclopropanes using both terminal and non-terminal alkenes (76–79). Mechanistically, upon photoexcitation, PC* reduced O_2_ to form a peroxide ion, O˙^−^, which abstracted an α-H from the active methyl compound 80, generating an α-C radical, 81 (Scheme 3c). The α-C radical then underwent an addition reaction with the alkene to form a secondary C radical (83), which reacted with O_2_ to produce a peroxyl radical anion 84. This radical anion subsequently extracted the α-H to form another α-C radical (85), which underwent the radical 1,3-substitution reaction with the peroxide to generate a cyclopropane ring. A distinctive aspect of this reaction was the application of iodine as a co-catalyst, derived either from molecular iodine or generated *in situ* from alkyl iodides.

**Scheme 4:**
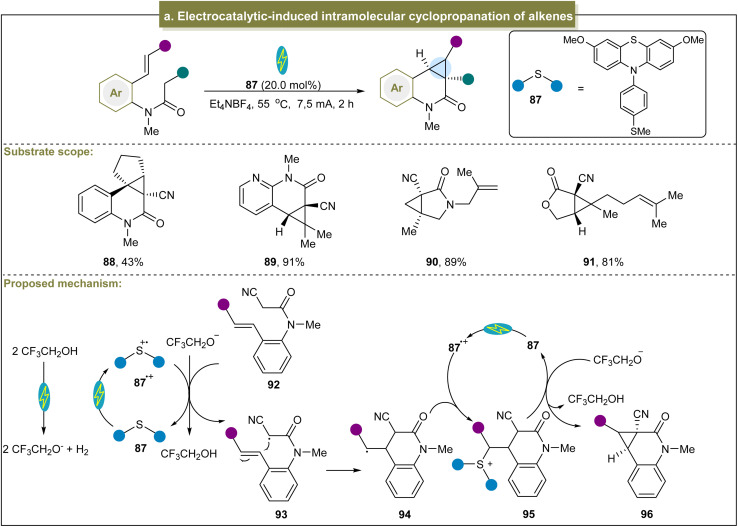
(a) Electrocatalytic-induced intramolecular cyclopropanation of alkenes.

Besides the above photoinduced strategies, Xu *et al.* achieved an efficient intramolecular cyclopropanation of active methylene compounds through electrocatalysis.^122^ With RVC (reticulated vitreous carbon) and Pt as electrodes and under constant current conditions, this reaction was compatible with cyclic or acyclic alkenes, providing desirable products in moderate to excellent yields (88 and 89). Furthermore, other reactive methylene compounds such as γ-lactams and γ-lactones also underwent smooth cyclopropanation (90 and 91). Mechanistically, the methylene substrate (92) was oxidized by 87˙^+^ to form an electrophilic carbon radical intermediate (93) (Scheme 4a). Subsequently, this radical attacked the alkene to obtain the alkyl radical (94) through cyclization. The alkyl radical then underwent radical–radical coupling with 87˙^+^ to yield the sulfonium ion 95. In the final step, nucleophilic substitution triggered by CF_3_CH_2_O^−^ in 95 formed the cyclopropanation product and regenerated 97. Unfortunately, this electrocatalytic system was only applicable to methylene compounds with an electron-withdrawing group such as the cyanide group.

Later, Koenigs *et al.* reported a method for synthesizing dialkyl-substituted cyclopropanes by the *in situ* generation of dialkyl diazo compounds from the corresponding *N*-tosylhydrazones.^123^ Based on the experimental and computational studies, the key step in this strategy involved the generation of α-Co(iii)-alkyl radicals from the activated dimethyl diazomethane. In addition, Pitre *et al.* described a method using vitamin B_12_ as a photocatalyst to facilitate cyclopropanation between dichloromethane and electron-deficient alkenes.^124^ This reaction was effective for a variety of functionalized, electron-deficient alkenes, yielding products in good to excellent yields under mild conditions. The method also utilized CD_2_Cl_2_ as a methyl source to prepare *D*_2_-cyclopropane products. Mechanistic studies have revealed that the Co(i) oxidation state of vitamin B_12_ can undergo S_N_2-type nucleophilic substitution with 1,1-dichloroalkane, followed by photolysis to generate a halogenated alkyl radical and a Co(ii) radical. The subsequent steps included Giese addition, single-electron reduction, and 3-*exo-tet* cyclization to produce the desired cyclopropane product. Because vitamin B_12_ has excellent biocompatibility and water solubility, this method is promising for the direct cyclopropanation of biomolecules.

The development of radical species for alkene reactions has proven effective and powerful for accessing cyclopropanes. Current radical precursors used in photo- and electrocatalysis include dibromides, carboxylic acids, and silicates. Moreover, adjacent electron-withdrawing groups can enhance the stability of carbon radical intermediates. The radical intermediates can then react with the alkene receptors to produce cyclopropanes. A variety of cyclopropane products can be successfully synthesized using these methods. However, ongoing efforts have focused on developing new radical precursors, utilizing accessible raw materials, and minimizing the reliance on costly catalysts.

### 3.2 Cyclopropanation through the construction of carbene/carbyne intermediates

**Scheme 5:**
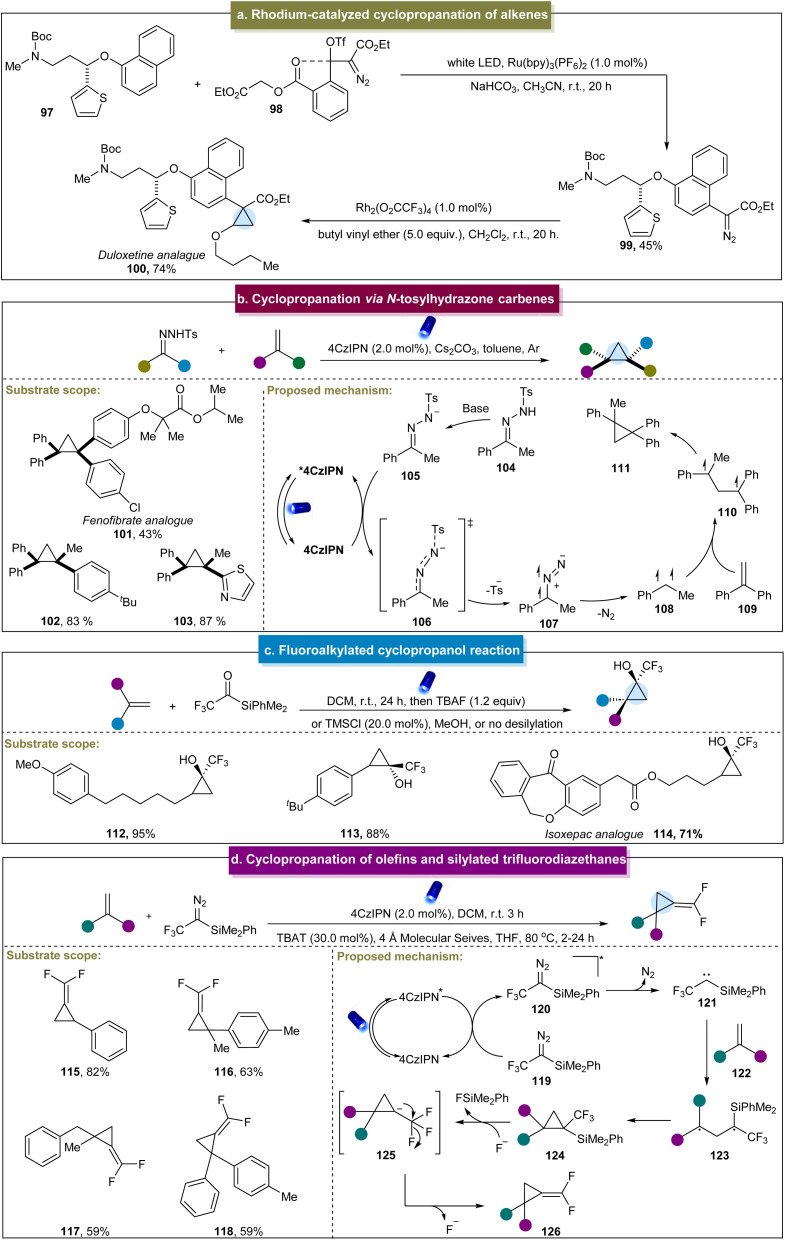
(a) Rhodium-catalyzed cyclopropanation of alkenes. (b) Cyclopropanation *via N*-tosylhydrazone carbene. (c) Fluoroalkylated cyclopropanol reaction. (d) Cyclopropane of alkenes and silylated trifluorodiazethane.

The carbene and carbyne intermediates have been widely adopted to synthesize cyclopropanes. In 2018, Suero *et al.* employed a photoredox strategy to generate diazomethyl radicals that served as direct carbyne equivalents (Scheme 5a).^73^ This approach facilitated the installation of tailored chiral centers at the carbon-hydrogen bonds, offering an efficient route to a potentially diverse library of bioactive molecules. However, this study was limited in substrate scope, focusing primarily on cyclopropanation, thereby highlighting further exploration in this field. Subsequently, Song *et al.* developed a method using aryl diazoacetates as carbene precursors under blue light irradiation, achieving the site-selective cyclopropanation of indole.^125^ This method accommodated a range of substituents on indoles and variations of diazo compounds. However, it did not apply to indoles with electron-donating properties or to unprotected indole compounds. Davies *et al.* developed a similar method enabling cyclopropanation with styrenes, indoles, pyrroles, and benzofurans, among others.^126^ They also utilized various aryl diazoacetates to yield cyclopropanes in moderate to excellent yields.

In 2020, Koenigs *et al.* developed another cyclopropanation method using diazoesters as the carbene precursors,^127^ achieving moderate-to-high yields with various methyl-substituted aromatic and polycyclic aromatic compounds. However, this strategy was limited by the application of donor–acceptor diazoalkanes and was unsuitable for benzene rings containing electron-withdrawing groups. The research team continued their research by investigating the cyclopropanation reactions between *N*-tosylhydrazones and indoles or thiophenes under stoichiometric conditions.^128^ This approach relied heavily on the donor–acceptor *N*-tosylhydrazones containing the electron-withdrawing groups as the carbene precursors. In 2020, Sen and co-workers reported the cyclopropanation of tryptamine derivatives to yield polycyclic indoles.^129^ However, the reactivity of the reaction was generally low, requiring additional protection of the indole substrates. Later, Koenigs *et al.* developed a method using aryl diazoacetates to cyclopropanate cyclooctadiene and polyunsaturated cyclic alkenes.^130^ This approach demonstrated broad applicability with various aryl diazoacetates and (poly)unsaturated cyclic hydrocarbons, efficiently synthesizing the functionalized bicyclic cyclopropanes in high yields and stereoselectivities. Shortly thereafter, they reported a continuous-flow method for generating cyclopropanes from aryl diazoesters and styrenes under photochemical conditions.^131^ In 2021, Barham *et al.* introduced another continuous-flow approach for the cyclopropanation of heterocyclic compounds.^132^ Compared to the traditional synthetic methods, continuous flow conditions can significantly improve the production efficiency and process safety in the large-scale experiments. Recently, Lv *et al.* presented a method using benzyl-substituted diazo esters as carbene precursors for the cyclopropanation of alkenes,^133^ which demonstrated excellent reactivity towards both aromatic and aliphatic substrates. Additionally, Xuan *et al.* reported the cyclopropanation of NHC boranes with diazo esters,^134^ achieving successful reactions with imidazole heterocyclic alkenes, while diazo esters containing electron-withdrawing groups on the benzene ring were unsuitable for this process.

In 2020, Wu *et al.* developed a photocatalytic method for the cyclopropanation of *N*-tosylhydrazones and aromatic alkenes in the presence of triethylamine.^135^ However, this approach has limited substrate compatibility, and the specific reaction mechanism is not detailed. Recently, Zhang *et al.* reported a visible-light-mediated strategy for synthesizing highly hindered cyclopropanes *via* energy transfer utilizing stable *N*-tosylhydrazones as accessible carbene precursors without hazardous diazo compounds.^75^ This method facilitated the straightforward construction of cyclopropanes in complex drug molecules and natural products. Initially, *N*-tosylhydrazone 104 underwent deprotonation to form the *N*-tosylhydrazone anion 105 in the presence of a base (Scheme 5b). Subsequently, the anion engaged in energy transfer with the excited-state catalyst 4CzIPN* to reach the triplet state (106), followed by the release of the sulfonyl group and nitrogen to generate triplet carbene species 108. Finally, the carbene species reacted with aromatic alkenes to complete the cyclopropanation process. Unfortunately, this strategy was only applicable to aromatic alkenes. Subsequently, König *et al.* developed an efficient method for the synthesis of spirocyclopropanes.^136^ Various alkyl-substituted *N*-tosylhydrazones reacted effectively with the electron-deficient alkenes in this two-step process, which could be readily terminated. Subsequent studies have suggested an ester substitution pathway involving a pyrazolidine intermediate. Recently, Wu *et al.* reported a strategy for selectively cyclopropanating 2-pyridones, which demonstrated high site selectivity and good tolerance towards functional groups.^137^ Although the mechanism was understood, this method could offer a versatile platform for the synthesis of bridged heterocycles.

In addition to the application of typical diazo esters or *N*-tosylhydrazones, Itoh *et al.* developed a synthetic method to generate selenium ylides from readily available dibenzoselenophenes.^138^ These ylides were excited under visible light to produce carbenes without the need for photocatalysts. This approach enabled the cyclopropanation of various alkenes through the formation of carbene species. However, the reaction involved the drawback of relatively low product yields. In 2022, the Priebbenow group described a reaction that generated a carbene intermediate from a ketone, followed by intramolecular [2 + 1] cyclopropanation with alkenes.^139^ Due to the high nucleophilicity of the silicon-based carbene intermediate, this method exhibited good compatibility with electron-deficient alkenes and could form spirocyclic frameworks, even with inactivated alkenes. The advantage of this strategy included the rapid reaction completion within 10 min without the requirement of a catalyst, while it required multiple complex reaction steps.

Furthermore, Shen *et al.* developed a strategy to directly synthesize fluoromethylated cyclopropanols using tri(di)fluoroacetylsilanes as carbene precursors.^74^ This reaction was suitable for various aliphatic and aromatic alkenes (Scheme 5c, 112 and 113). Moreover, the gram-scale reaction and late-stage functionalization of complex molecules, such as isoxepac (114), indicated the synthetic capabilities of this method. The mechanism involved a three-linear-state carbene, rather than a synergistic mechanism with a single-linear-state carbene. Although the strategy was novel, this approach did not extend to non-terminal alkenes, and the selectivity of difluoromethyl acylsilane-derived carbenes was generally lower than that of trifluoromethyl acylsilane-derived carbenes in reactions. Later, they continued their exploration in this field and reported the synthesis of (difluoromethylene) cyclopropanes *via* the reaction of silylated trifluorodiazoethanes with aromatic alkenes.^140^ This one-pot strategy exhibited excellent functional group tolerance, with the ability to tolerate various aromatic and aliphatic alkenes, yielding a diverse range of (difluoromethylene) cyclopropanes (115–118) in moderate to excellent yields. Based on the detailed mechanistic experiments and computational data, a plausible mechanism was proposed (Scheme 5d). Initially, the excited state of the catalyst underwent energy transfer (EnT) with 119, generating 120 and subsequently releasing N_2_ to form triplet carbene 121. This triplet carbene was added to the alkene to form a diradical intermediate (123), which then underwent intramolecular cyclization to generate cyclopropane product 124. Fluoride then attacked the silyl group, generating unstable anionic intermediate (125), which finally eliminated fluoride to yield (difluoromethylene) cyclopropane. In 2023, Gryko *et al.* used 1,3,4-oxadiazole compounds as diazo precursors for the synthesis of spirocyclopropanes.^141^ The excited catalyst transferred the energy to the 1,3,4-oxadiazole compound in its triplet state. Subsequently, the cleavage of the C_2_–N_3_ bond in the oxadiazole led to the formation of a diazo group, which decomposed into the triplet carbene and diazoalkane. In the presence of alkenes, the triplet carbene formed a diradical intermediate followed by cyclopropane. Simultaneously, diazoalkanes underwent 1,3-dipolar cycloaddition with an alkene, resulting in the formation of pyrazoline. The pyrazoline could generate the spirocyclopropane *via* the diradical intermediate state under photosensitization. Although this strategy was effective for synthesizing various alkyl-substituted spirocyclopropanes, it was challenging to synthesize aromatic cyclopropanes that did not react with alkenes containing electron-donating groups.

Overall, photo/electrochemical methods have recently emerged as effective approaches for accessing carbene/carbyne intermediates. These mild and efficient techniques have enabled several carbene precursors, including diazo compounds, *N*-tosylhydrazones, and ylides to effectively facilitate cyclopropanation, significantly advancing the field of photocatalytic carbene chemistry. Despite the substantial progress made with these strategies, safer and more widely available carbene precursors still need to be designed, especially compared to cyclopropanation *via* radical processes.

### 3.3 Cyclopropanation through the activation of alkenes

**Scheme 6:**
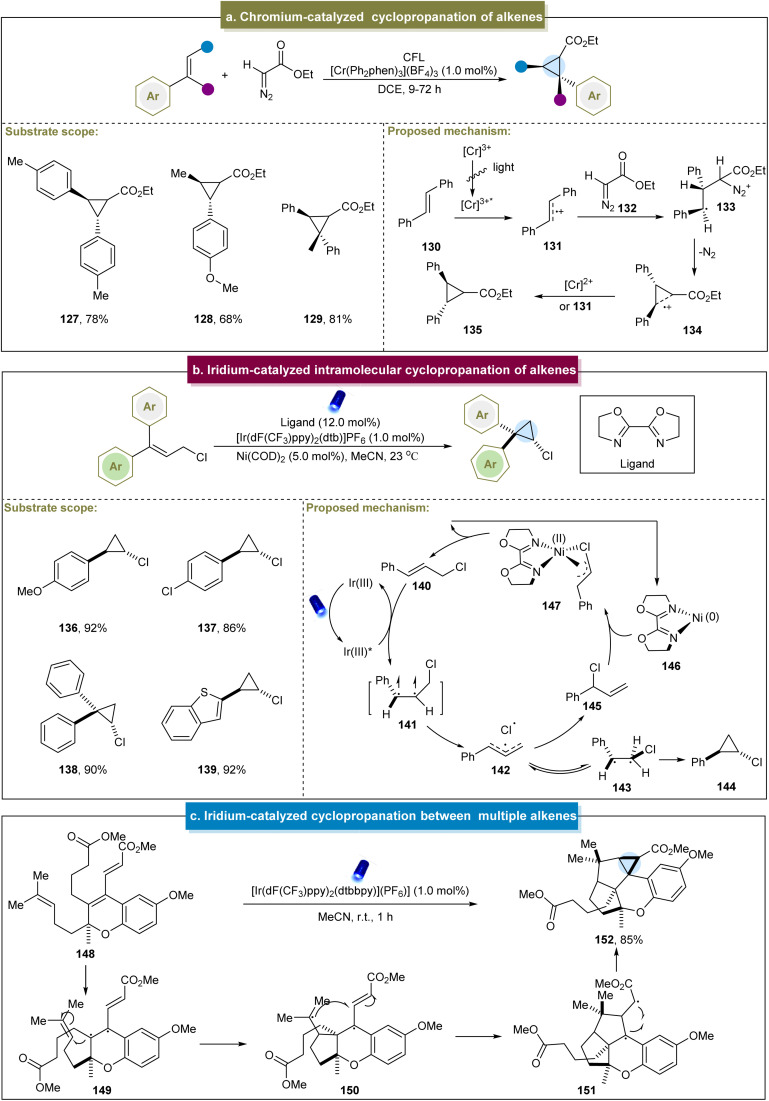
(a) Chromium-catalyzed cyclopropanation of alkenes. (b) Iridium-catalyzed intramolecular cyclopropanation of alkenes. (c) Iridium-catalyzed cyclopropanation between multiple alkenes.

In addition to the significant efforts to access cyclopropanes *via* radical or carbene pathways, the activation of alkenes to produce cyclopropanes is another crucial approach. In 2017, Ferreira *et al.* disclosed a visible light-mediated cyclopropanation between electron-rich aromatic alkenes and diazoesters.^76^ A range of aryl alkenes were successfully applied to cyclopropanation under standard conditions (127–129). For the cycloaddition reaction, the excited state Cr(iii) catalyst initially induced a single-electron oxidation of alkene (130), and the resulting radical cation (131) reacted with the nucleophilic diazo compound (Scheme 6a). The loss of N_2_ provided a “long-bonded” radical cation intermediate 134. At this stage, electrons were transferred from either the reduced-state Cr complex or another equivalent of the alkene to produce the cyclopropane product. However, this approach still faced challenges, such as its inapplicability to tetrasubstituted alkenes and the nullification of the reaction by some arylalkyl alkenes, which requires further exploration.

Later, Zeller *et al.* developed a photo-induced method for the intramolecular cyclopropanation of alkene–alkyne molecules.^142^ The initial step of this reaction involved the oxidation of the alkyne by an excited-state photocatalyst to form an ynamide radical cation, followed by a radical cascade process to complete the cyclopropanation. This strategy provided a valuable new approach for the synthesis of various 3-aza[*n*.1.0]bicycles.

In 2020, Tambar *et al.* reported the photocatalytic intramolecular cyclopropanation of acyclic cinnamyl chlorides.^77^ The reaction exhibited high tolerance towards the aromatic systems with various substituents and demonstrated excellent anti-selectivity and yields (136–138). This approach also indicated the tolerance to heterocycles such as benzothiophene (139). Upon irradiation, the energy transfer from the Ir(iii) photocatalyst activated the cinnamyl chloride (140) to its excited state (141, Scheme 6b). Subsequently, a triallyl pair (142) containing a chlorine radical was formed from the excited complex. There were two pathways for intermediate 142. One path passed through triplet-β-Cl intermediate (143), generating the desired cyclopropanation product through radical coupling. The other pathway involved the reaction of Ni(0) complex (146) to form the nickel alkenyl complex (147), thus regenerating cinnamyl chloride. Unfortunately, this strategy was not applicable to cyclopropanation of terminal alkenes. Similarly, Schindler *et al.* reported a 14-step synthesis of (+)-cochlearol B, suggesting that the cyclopropane byproduct was obtained during the photochemical reaction (Scheme 6c).^143^ In this process, the alkene in 148 was initially excited to its photochemical state, generating the diradical 149. The resulting diradical reacted with the homoprenyl subunit to form ring B (150). Subsequently, addition to the electrophilic carbon of the methyl acrylate fragment occurred, resulting in a second five-membered ring (151). Ultimately, this led to the formation of cyclopropanes through radical recombination.

**Scheme 7:**
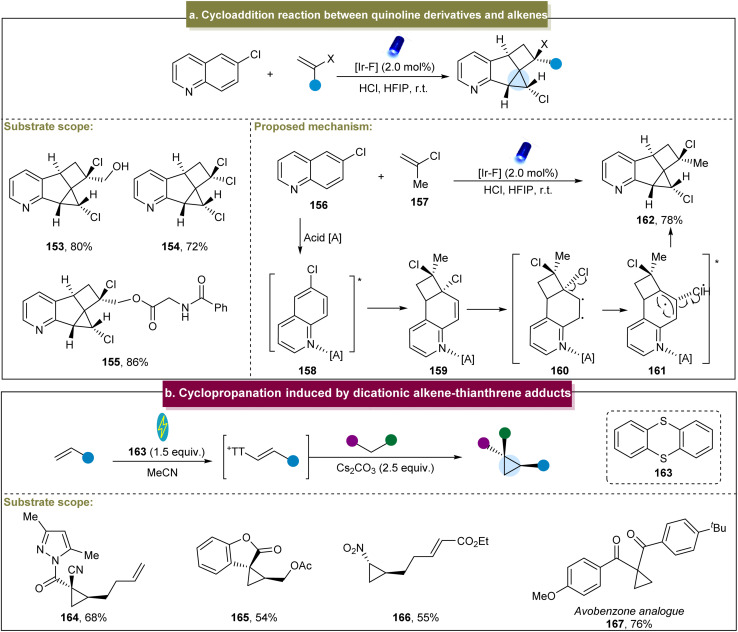
(a) Cycloaddition reaction between quinoline derivatives and alkenes. (b) Cyclopropanation induced by the dicationic alkene–thianthrene adduct.

Recently, Glorius *et al.* reported an energy transfer-mediated cascade dearomatization [2 + 2] cycloaddition between the quinoline derivatives and aromatic alkenes, selectively yielding the corresponding cyclopropane.^144^ The method exhibited excellent tolerance towards diverse functionalized 2-chloropropene derivatives, providing the corresponding chlorinated cyclopropanes in good yields (153–155). A plausible mechanism was proposed (Scheme 7a). Initially, an energy transfer occurred between 156 and the catalyst, generating the excited state 158. Subsequently, 158 underwent [2 + 2] cycloaddition with 157 to form the cinnamyl chloride intermediate 159. Intermediate (159) underwent another energy transfer, leading to the cleavage of the C–Cl bond and generation of triplet radical pair 161. Finally, the Cl radical reacted with the intermediate radical to form the C–Cl bond, and the radicals at both ends couple to form the C–C bond, yielding the final cyclopropanes. This energy-transfer-mediated strategy demonstrated remarkable *cis*-selectivity, circumventing the general reactivity and selectivity issues associated with the photochemical [2 + 2] cycloadditions of various other aromatic substances.

In 2023, Wickens and co-workers developed a modular method for the preparation of cyclopropanes.^78^ With RVC and Ni as electrodes and under constant current conditions, this method was scalable and applicable for the generation of diverse cyclopropanes and exhibited broad substrate tolerance including diverse alkenes and nucleophilic reagents (Scheme 7b, 164–167); however, this method was restricted to specific alkenes bearing electron-withdrawing groups. Mechanistic studies have indicated that the dicationic addition compound was rapidly eliminated under the reaction conditions to form the alkenyl thianthrenium salt, a crucial electrophilic intermediate in the cyclopropanation reaction. Compared with the traditional metal-catalyzed methods, this approach has expanded the utilization of inexpensive coupling agents and readily available reagents for constructing the cyclopropane building blocks.

In summary, strategies for activating alkenes in the synthesis of cyclopropanes through photo/electrocatalysis mainly involve generating the alkene radical cations or diradicals that efficiently enable diverse cyclopropane syntheses. However, the photoredox activation of alkenes is generally challenging because of their relatively high redox potentials. Therefore, the selectivity of alkene activation should be addressed when applying this method.

### 3.4 Cyclopropanation through other methods

In addition to the aforementioned methods for synthesizing cyclopropanes *via* donor- or acceptor-induced reactions, other methods can be triggered by the exceptional reactivity. For instance, Egorov *et al.* developed an electrochemical method for the synthesis of cyclopropanes from alkylidene malononitriles and malononitriles.^145^ This method differed from the radical generation through halogen departure. The presence of pyridine facilitated the generation of carbanion intermediates upon the departure of the bromine atom.

**Scheme 8:**
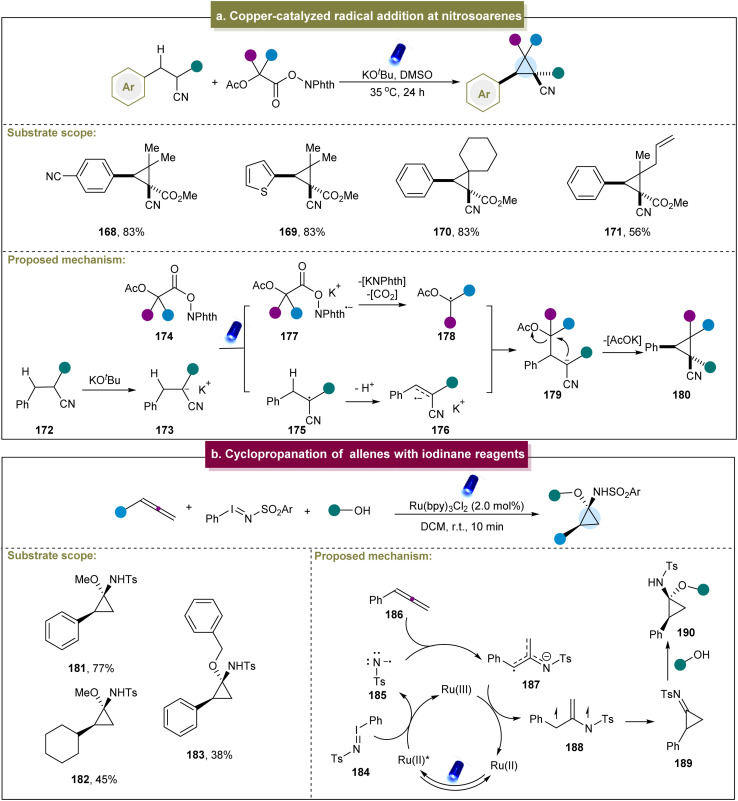
(a) Iridium-catalyzed intramolecular cyclopropanation of alkenes. (b) Cyclopropanation of allenes with iodinane reagents.

In 2021, Hayashi *et al.* introduced a photosynthetic method that directly coupled the cyanoalkanes with electron-accepting C1 carbon units to produce cyclopropanes.^93^ This method exhibited excellent chemical selectivity for both electron-rich and electron-poor para-substituted phenyl groups, yielding cyclopropanes in good yields. Subsequently, a possible mechanism for this strategy was proposed (Scheme 8a). First, under the influence of a base, the cyanoalkane underwent deprotonation to generate the potassium salt 173. The α-anion (173) formed an electron donor/acceptor complex with *N*-hydroxyphthalimide ester (174). Under light irradiation, this complex generated the β-acidic α-radical (175) and radical anion (177). The radical anion (177) fragmented into a C1 α-radical (178) by releasing CO_2_ and *N*-phthalimide anions. The α-radical (175) lost the β-proton to obtain the α/β-radical anion (176). The intermolecular homogeneous coupling between the radical anion and the *o*-stabilized radical then formed a new C_sp^3^_–C_sp^3^_ bond at the β-position, resulting in the cyclization of the α-anion (179) to provide the corresponding cyclopropane. However, this strategy had some limitations; for example, the redox-active ester had poor tolerance towards aromatic rings, and the cyano group and strong electron-withdrawing groups were also necessary. Similarly, Kawashita *et al.* developed an electrochemical transformation method to convert alkyl 2-chloroacetates into cyclopropanes.^146^ Although the substrate range for this reaction was relatively limited, it offered a new approach for preparing 1,2,3-trisubstituted cyclopropane derivatives under electrochemical conditions.

Furthermore, Koenigs *et al.* reported a photocatalyzed cyclopropanation of allenes using iodinane reagents.^94^ Exploration of the substrate scope revealed that various aromatic allenes with different functional groups efficiently produced amino cyclopropanes in high yields, while aliphatic allenes showed decreased reactivities (181–183). Finally, a plausible mechanism for this reaction was proposed (Scheme 8b). Initially, iodinane 184 underwent the SET with the photo-excited catalyst, generating the nitrene radical anion 185, which was added to the allenic carbon atom to form 187. Subsequently, 187 underwent further oxidation with the ruthenium catalyst, followed by cyclization, leading to the formation of the highly reactive cyclopropyl imine intermediate 189. The experimental results indicated that the cyclopropyl imine intermediate could react with methanol to form the cyclopropyl amine product. Jubault *et al.* reported a method for electrochemically generating cyclopropylamines from the corresponding cyclopropyl amides, primarily using the Hofmann rearrangement strategy.^147^

In summary, distinct from the radical, carbene, and alkene initiation pathways for cyclopropane construction, there were still intriguing and promising strategies under photo/electrocatalytic conditions, such as EDA complex formation or direct photolysis. These approaches could facilitate the design of simpler and greener cyclopropanation methods.

## 4 Synthesis of aziridines by photo/electrochemical approaches

In recent years, significant advancements have been achieved in the synthesis of aziridines using photo/electrochemical methods.^79^ These include donor-initiated strategies that utilize nitrenes, nitrene radical anions, *N*-radicals, *N*-iodoamines, and formaldimines to selectively produce aziridines from various alkenes.^80,81^ Additionally, acceptor-initiated approaches involving alkene radical cations and dicationic alkene–thianthrene adducts have been developed.^82^ These methodologies have been applied diversely, including the aziridination of styrene derivatives, coumarins, and aliphatic amines.^83–85^

### 4.1 Aziridination through donor-initiated methods

**Scheme 9:**
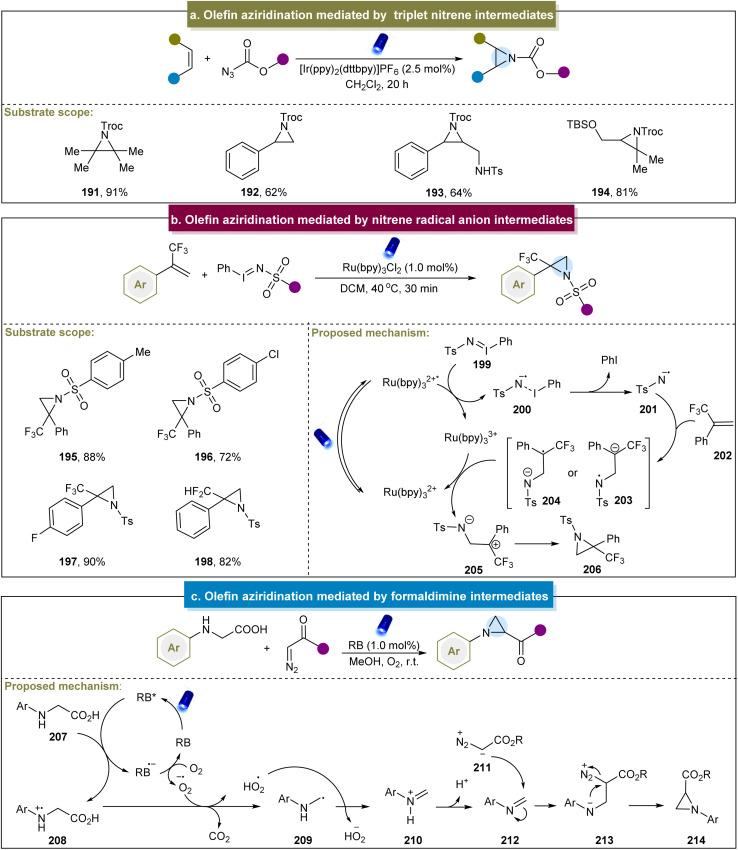
(a) Alkene aziridination mediated by a triplet nitrene intermediate. (b) Alkene aziridination mediated by a nitrene radical anion intermediate. (c) Alkene aziridination mediated by formaldimine intermediates.

In 2016, Yoon *et al.* documented a visible-light-mediated aziridination method that selectively formed triplet nitrenes from azidoformates.^79^ Mechanistic studies have confirmed that the aziridination was mediated through the [3 + 2] cycloaddition of the carbamoyl azide, followed by the photocatalytic decomposition of the 1,2,3-triazoline intermediate. Particularly, diverse alkenes including styrenes, aliphatic alkenes, tetrasubstituted alkenes, *etc*. were accommodated in this system (Scheme 9a, 191–194). Furthermore, the reaction could be conducted on a preparative scale by using a flow reactor. However, there have been no reports of aromatic alkenes containing electron-donating groups, and no explicit mechanism has been reported. Inspired by this elegant work, the group of Lu developed a selective intramolecular *o*-allylphenyl azidoformate aziridination through an energy transfer mechanism.^148^ This method involved the generation of triplet nitrene through the release of N_2_ by triplet azides. The triplet nitrene then undergoes radical addition with an alkene, followed by low-energy barrier radical coupling to yield the desired aziridine product. A limitation of the manuscript was the lack of in-depth exploration regarding the introduction of allyl groups. Similarly, Zhu *et al.* developed catalyst-free visible-light-induced alkene aziridination.^149^ This reaction proved effective for the terminal alkenes, intramolecular alkenes, electron-deficient alkenes, and dienes. However, the system suggested the intolerance towards heteroatom-containing alkenes and electron-donating groups.

Furthermore, Koenigs *et al.* reported the direct aziridination of iodinane with α-trifluoromethyl styrenes under photocatalytic conditions.^80^ This strategy utilized iminoiodinanes to generate nitrene radical anions (200) *via* the oxidative quenching of the excited state of Ru(bpy)_3_^2+^ (Scheme 9b). The nitrene radical anion proved to be a versatile intermediate, enabling the synthesis of fluorinated aziridines, including perfluoroalkyl and difluoromethyl aziridines (195–198). However, alkylsulfonyl groups were incompatible under these conditions, and limitations were observed with *ortho*-substituted aromatic rings, pyridine, and aliphatic alkenes. Building on Königs's work, Rastogi *et al.* reported a photoredox aziridination strategy for synthesizing chalcones.^150^

What's more, Zhou and co-workers developed a decarboxylative aza-Darzens reaction to synthesize monosubstituted aziridines *via* formaldimine intermediates.^83^ The reaction mechanism, depicted in Scheme 9c, began with the quenching of the excited state RB* by *N*-aryl glycine (207) to form cationic radical 208. Subsequent decarboxylation of 208 generated the α-amino alkyl radical (229), which underwent oxidation by superoxide radicals to form iminium ion 210. The deprotonation of the iminium ion produces the active imine 212, and aziridine formation occurred through the nucleophilic addition of diazo compounds to the C=N bond, followed by intramolecular nucleophilic attack of the nitrogen atom with N_2_ as the leaving group. More recently, Xuan *et al.* introduced a solvent-controlled divergent cycloaddition reaction for the construction of aziridines using α-diazo esters with hexahydro-1,3,5-triazines.^151^ Mechanistic studies have identified formaldimine as the key intermediate in this strategy. Generally, the activation of nitrogen atoms in donor compounds and their reactions with alkenes represent straightforward and efficient approaches for obtaining aziridines. Current aziridination strategies using photo/electrocatalysis primarily involve nitrenes, nitrene radical anions, *N*-radicals, *N*-iodoamines, and formaldimines.

**Scheme 10:**
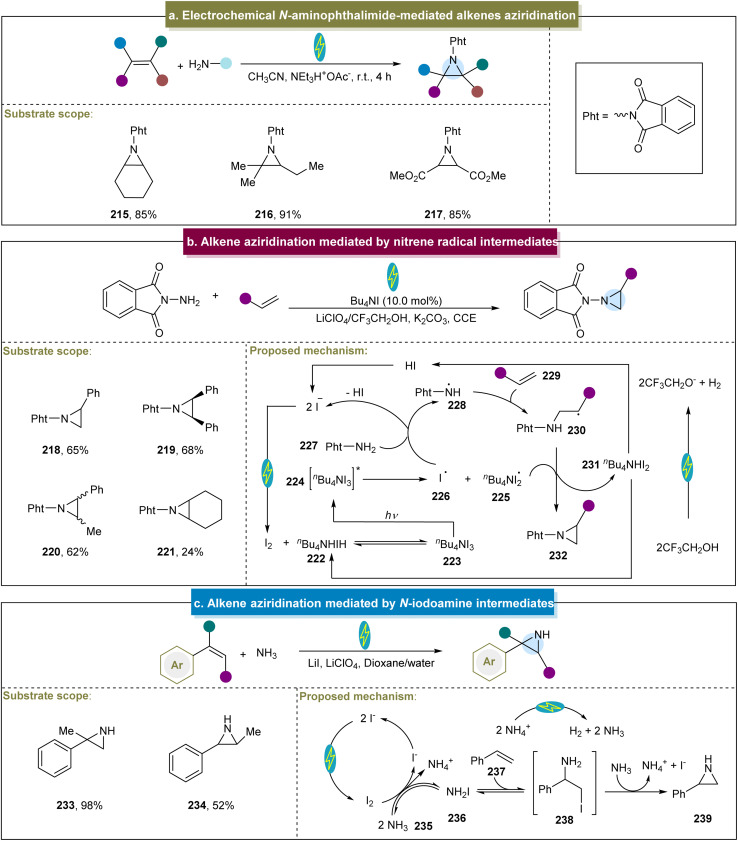
(a) Electrochemical *N*-aminophthalimide-mediated alkene aziridination. (b) Alkene aziridination mediated by a nitrene radical intermediate. (c) Alkene aziridination mediated by an *N*-iodoamine intermediate.

In addition, Siu and Yudin outlined an electrochemical aziridination method employing *N*-amino phthalimide as the nitrogen source.^152^ This approach offers the notable advantage of eliminating the need for stoichiometric amounts of toxic oxidants and metal additives. Moreover, with platinum as the electrode and under constant voltage conditions, both electron-rich and electron-poor aliphatic alkenes were efficiently converted to aziridines (Scheme 10a, 215–217). However, this study could not offer a detailed explanation of this mechanism.

In addition to the nitrene and nitrene radical anion methods, another group developed an efficient electrochemical aziridination strategy utilizing *N*-radicals as key intermediates.^81^ In this case, with GC (graphitic carbon) and iron as electrodes and under constant current conditions, a wide range of aromatic or aliphatic alkenes had been proved to be compatible with the catalytic system (218–221). However, the reactivity of aliphatic cyclic aromatic hydrocarbons under these reaction conditions was poor, indicating room for further improvement. Initially, the anodic oxidation of iodide generated molecular iodine, which reacted with *n*-Bu_4_NI to form ammonium triiodide (*n*-Bu_4_NI_3_, 223, Scheme 10b). Under visible-light irradiation, I˙ (226) was produced and abstracted a hydrogen atom from Pht-NH_2_ (227), forming the aminyl radical 228. This radical then reacted with the alkene to form radical (230). The homolytic cleavage of the N–H bond in 230, facilitated by I_2_˙^−^ through hydrogen atom abstraction, was followed by intramolecular cyclization, yielding the aziridine products. Subsequently, Itoh *et al.* reported a practical aziridination method using the styrene derivatives and *p*-toluenesulfonamide as the nitrogen source.^153^ Various functionalized styrenes were adopted in this system, affording the aziridines in excellent yields, whereas the aliphatic alkenes did not react. The proposed mechanism involved TsNH_2_ reacting with molecular iodine to form the intermediate TsNHI. The photolysis of the N–I bond then generated a nitrogen-centered radical that reacted with the styrene to form the target aziridine. Similarly, the group of Xu developed a method for achieving the stereospecific aziridination of alkenes through photoredox catalysis.^154^ Aziridines were synthesized using various alkenes and *N*-protected aminopryridinium salts as the nitrogen radical sources, achieving excellent stereocontrol. However, the reaction conditions were not suitable for alkenes containing vinyl groups with electron-donating substituents.

Additionally, other methods have been developed for the construction of aziridines using *N*-iodoamine intermediates. He *et al.* initially reported the electrochemical aziridination of chalcones using simple amines as the nitrogen sources.^155^ Unfortunately, anilines and non-chalcone-type alkenes were not suitable for this approach. Furthermore, Cheng *et al.* described the ArI-mediated electrochemical aziridination of electron-deficient alkenes in an undivided cell.^82^ The reaction proceeded through a stepwise pathway involving *in situ*-generated iodine nitrene and acetyl hydrazine as the aziridination reagents. This method enabled a scale-up to 10 g, and the product served as a precursor for α-amino acids. Recently, De Vos *et al.* utilized inexpensive ammonia as the nitrogen source and iodide as a redox mediator for N–H aziridination (Scheme 10c).^156^ Using carbon and Ni as electrodes under constant current conditions, this work generated a series of unprotected N–H aziridines without the need for pre-oxidized nitrogen sources or subsequent organic de-protection steps, demonstrating the excellent atomic economy. Moreover, mechanistic studies have indicated that straightforward N–I species, such as NH_2_I, are effective tools for aziridination.

### 4.2 Aziridination through the activation of alkenes

**Scheme 11:**
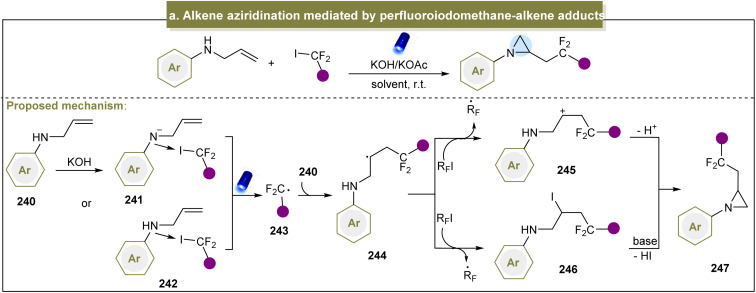
(a) Alkene aziridination mediated by a perfluoroiodomethane–alkene adduct.

Considerable efforts have been devoted to exploring pathways for accessing aziridines through donor-initiated methods. The activation of alkenes to facilitate aziridination has emerged as a crucial approach. In 2013, Cho *et al.* developed a visible-light-induced trifluoromethylation of allylamines to synthesize CF_3_-containing aziridines, emphasizing selective nucleophilic reactions over intermediate elimination reactions.^87^ Moreover, perfluoroalkylations with other perfluoroalkyl iodides such as C_3_F_7_I and C_4_F_9_I proceeded efficiently under standard conditions. Subsequently, Zhao *et al.* introduced a strategy for direct fluorinated aziridination of alkenes, leveraging the non-covalent interactions between *N*-allylanilines and fluoroalkyl iodides.^85^ However, the use of heteroaromatic substrates was not suitable for this reaction, limiting its application in practical synthesis. The potential mechanism of this reaction is shown in Scheme 11a. Initially, a non-covalent interaction occurred between amine (240) and the C–I bond. Upon irradiation with visible light, the carbon radical (243) was generated. The subsequent cyclization proceeded *via* two possible pathways: (1) the resulting radical (244) abstracted an iodine atom from R_F_I, forming an intermediate (245). Finally, the desired aziridine was obtained in the presence of a base *via* additional cyclization reactions. (2) Intermediate (245) was formed *via* the SET process between 244 and R_F_I, leading to the generation of the desired product (247) through subsequent cyclization reactions.

**Scheme 12:**
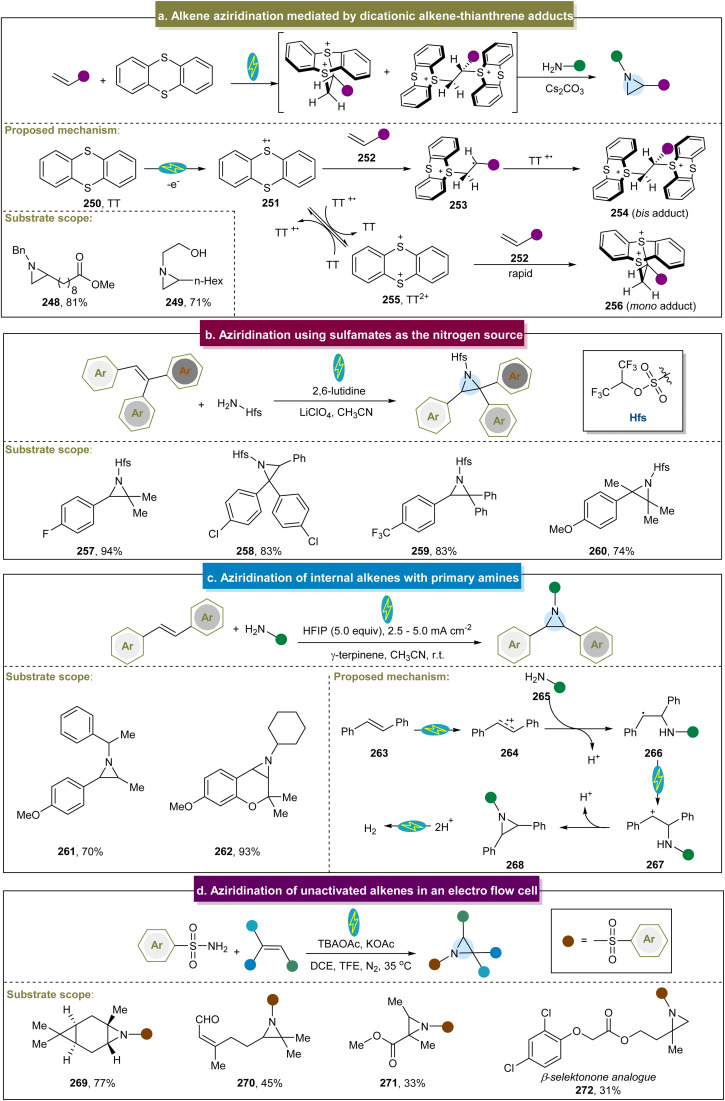
(a) Alkene aziridination mediated by a dicationic alkene–thianthrene adduct. (b) Aziridination using sulfamates as the nitrogen source. (c) Aziridination of internal alkenes with primary amines. (d) Aziridination of unactivated alkenes in an electro flow cell.

Recently, Wickens *et al.* identified an electrochemical strategy to obtain a metastable dicationic intermediate from aliphatic alkenes.^84^ With RVC as the electrode and under constant current conditions, the experiments demonstrated that the aziridine products were obtained from a broad range of amine nucleophiles, significantly broadening the limited scope of feasible *N*-substituents in aziridines synthesized from alkenes using traditional electrophilic nitrogen reagents (Scheme 12a, 248 and 249). Unfortunately, intramolecular and aryl alkenes were not suitable in this case, and the method still did not address the challenge of unreactive aromatic amines. The proposed reaction mechanism indicated that the mono- and bis-TT dicationic adducts (254 and 256) could be formed in high yields by reacting terminal alkenes and thianthrene (250) under the optimized electrolytic conditions. By simultaneously adding a primary alkylamine and an inorganic base without further separation, both cationic species were consumed to yield the desired aziridine products. In 2018, Li *et al.* developed an electrochemical strategy for generating aziridines using sulfamates as the nitrogen source.^157^ With the use of graphite felt electrodes and the condition of constant voltage, the direct aziridination of triaryl substituted alkenes was realized for the first time. Even though this electrochemical reaction was extended to use other multisubstituted alkenes, aliphatic alkenes were not feasible in this system (Scheme 12b).

Noël *et al.* proposed an electrochemical method for preparing aziridines through oxidative coupling between alkenes and primary alkylamines (Scheme 12c).^158^ With the use of carbon and Fe as electrodes, and under constant current conditions, this conversion was effectively conducted using primary alkylamines and alkenes with various structures and electronic properties (261 and 262). These findings indicated that the anodic oxidation of the alkene initiated the electrochemical aziridination process. Upon formation of the radical cation (274), it readily reacted with the amine (265) coupling partner. Following the deprotonation and subsequent SET, a carbocation (267) formed and underwent rapid, barrier-free intramolecular ring closure and deprotonation to yield the desired aziridine product. Unfortunately, electron-withdrawing functional groups and heterocyclic aromatics were not amenable, and this strategy didn't involve terminal alkenes and aromatic amines. Recently, Lei *et al.* introduced an electro-oxidative flow method that successfully achieved aziridination of natural products without requiring additional oxidants (Scheme 12d), and the flow cell was equipped with carbon paper, a platinum plate was used as the cathode and electrolyzed at a constant current.^159^ Two possible mechanisms were proposed for this reaction. The first pathway involved the initial oxidation of alkenes to form alkene radical cations, which then react with sulfonamides. The second pathway involved the simultaneous oxidation of sulfonamides and alkenes, followed by polar cross-coupling to produce the desired product. This strategy had a wide range of aliphatic substrates (269–271), and a series of aziridine derivatives from bioactive molecules were synthesized efficiently in good yields including 272.

The synthesis of aziridines using photo/electrocatalytic methods has attracted considerable attention, particularly for electrocatalytic applications. Numerous strategies have been proposed for providing diverse methodological options. However, certain methods are constrained by specific substrates or functional groups, limiting their applicability. Furthermore, there are relatively few reports on the photoinduced activation of alkenes for aziridination. Therefore, there is a pressing need to develop more universal and efficient methods to overcome substrate limitations and meet the broader demands for aziridine syntheses.

## 5 Synthesis of epoxides by photo/electrochemical approaches

The formation of epoxides often involves the participation of O_2_, which can be converted into ^1^O_2_ or O_2_˙^−^ to facilitate epoxide formation. Additionally, epoxides can be generated by reacting alkenes with perfluoroiodomethane–alkene adducts, followed by the reactions with hydroxyl groups.^87^ Furthermore, the advancements in epoxide production have significantly expanded renewable energy applications,^160–162^ as summarized by Banerjee *et al.* in the electrocatalytic synthesis.^163^ Therefore, our focus was on the synthesis of small molecules containing epoxides, with a discussion of both the classic methods and recent advancements.

**Scheme 13:**
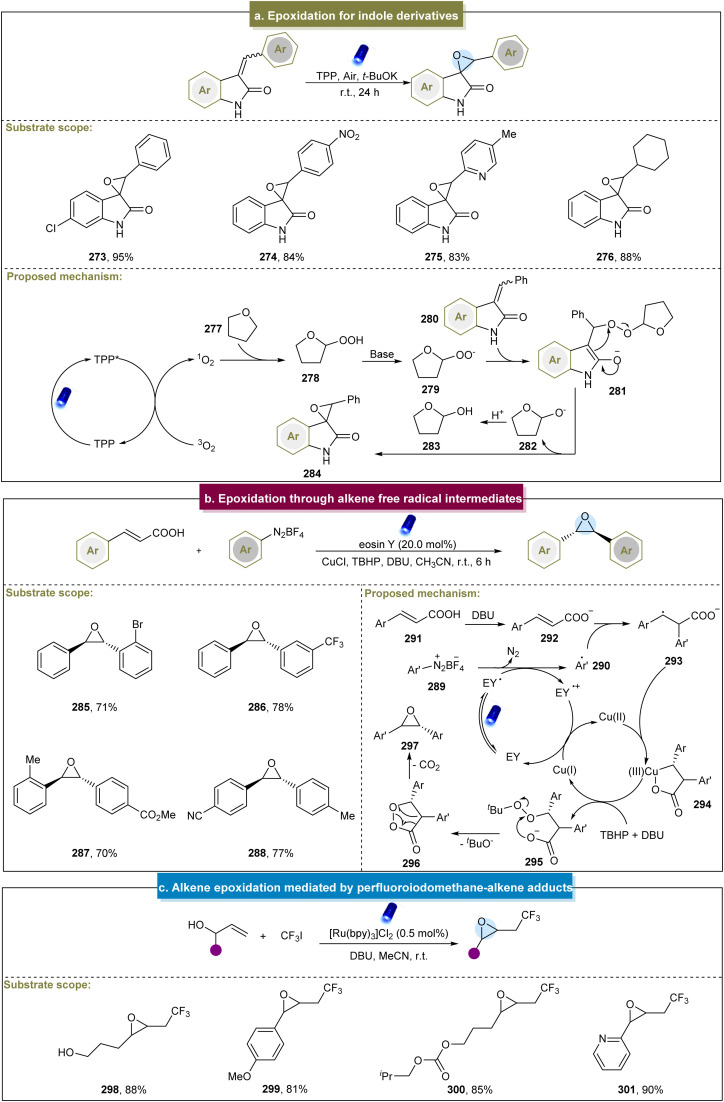
(a) Epoxidation through alkene free radical intermediates, TPP (*meso*-tetraphenylporphyrin). (b) Pd/ZnO nanoparticles catalyze epoxidation of alkenes. (c) Alkene epoxidation mediated by a perfluoroiodomethane–alkene adduct.

Itoh *et al.* (2009) developed a convenient method for the epoxidation of various alkenes under fluorescent lamp irradiation.^164^ This method proved effective for aromatic-substituted alkenes, alkyl-substituted internal alkenes, and terminal alkenes but was unsuitable for alkyl-substituted alkenes. Subsequently, Katsuki *et al.* suggested that the Ru(NO)-salen complex could epoxidize aromatic alkenes under visible-light irradiation.^165^ However, this approach did not work well with substrates containing electron-withdrawing functional groups, and the mechanism remained unclear. Similarly, Luo *et al.* reported the visible-light-induced epoxidation of α,β-unsaturated ketones.^166^ The experimental and DFT calculations supported a mechanism involving the single linear oxygen. This method yielded a range of aromatic/aliphatic α,β-epoxy ketones but required long reaction times, which could be mitigated by employing the flow reaction. The reaction required precise temperature control, and compounds containing amide substituents were incompatible under these conditions. Later, Jin *et al.* discovered a visible-light-induced aerobic epoxidation method for the synthesis of spiro-epoxyoxindole derivatives (Scheme 13a).^167^ They extensively explored the substrate scope of the two aromatic rings, achieving smooth reactions and synthesizing target compounds in moderate to excellent yields. However, the presence of various substituents at different positions on 3-benzalindolin-2-ketone affected the efficiency of the reaction, and there has been limited exploration of aryl heterocycles as substrates under these conditions. In 2022, the Coeffard group described a photocatalytic method for epoxide synthesis.^168^ The reaction likely involved the singlet oxygen in oxidative dearomatization to form a hydroperoxide intermediate, followed by intramolecular oxygen atom transfer to the alkene side chain to yield epoxides.

In 2020, Huang *et al.* reported an epoxidation strategy using pyrenediones as the photocatalysts.^169^ This system proved effective for various aromatic and aliphatic alkenes. However, a thorough exploration of the reaction mechanism is lacking. Subsequently, Chao *et al.* described an epoxidation catalyzed by acetylated vitamin B_2_, targeting α,β-enones formed *in situ via* major SET and minor energy transfer pathways.^170^ This catalytic system successfully epoxidized a range of electron-deficient alkenes. However, it was incompatible with heterocyclic alkenes, and the reaction of α,β-enones containing sequences of aryl and aliphatic groups was unsatisfactory.

Recently, Singh *et al.* synthesized *trans*-oxirane oxide using TBHP as an oxidant.^86^ Various cinnamic acids and aryl diazonium salts reacted effectively (285–288). However, some aliphatic/heteroaromatic α,β-unsaturated carboxylic acids did not yield the desired products. The proposed mechanism is illustrated in Scheme 13b. Initially, the aryl diazonium salt (289) and excited eosin Y (EY*) underwent the SET process to form the aryl radical (290). The resulting interaction between the aryl radical and cinnamate (292) formed an intermediate (293), which chelated with Cu(ii) to form a five-membered ring (294). In the presence of TBHP and DBU, this chelated ring proceeded through intermediate (295) to generate another species, 1,2-dioxolan-3-one (296), which ultimately underwent decarboxylation to produce the desired epoxides.

In addition to the ^1^O_2_ or O_2_˙^−^ pathways, Cho *et al.* developed a method in 2013 for the visible-light-induced trifluoromethylation of allylic alcohols. This method involved a selective nucleophilic reaction *via* elimination of an intermediate to yield CF_3_-containing epoxides.^87^ Mild reaction conditions made this possible for a series of inactivated alkenes with different functional groups (Scheme 13c, 298–301). Unfortunately, this strategy has not yet been applied to the epoxidation of internal alkenes. Itoh *et al.* described the one-pot synthesis of α,β-epoxy ketones using benzyl alcohols and styrenes.^171^ This method involved the simultaneous oxidation of benzyl alcohols and styrenes to form benzaldehydes and phenacyl iodides, followed by the Darzens reaction to produce epoxides. Additionally, there was room for improvement, particularly because the styrenes with electron-donating groups exhibited poor reactivity. Furthermore, De Vos *et al.* introduced an electrocatalytic method for epoxidation.^172^ This approach achieved high yields with various aromatic- and aliphatic-substituted alkenes. Mechanistic studies have revealed that Br_2_ first formed Br^+^ species with alkenes under electric influence. Subsequently, under alkaline conditions, 2-bromoalkanol compounds were generated and the final products were obtained by eliminating bromide.

Various strategies have been developed for synthesizing epoxides, primarily involving the generation of superoxide intermediates to attack alkenes for epoxidation or to cyclize through the attack of neighboring leaving groups by hydroxyl groups. However, the existing literature often limits the substrate scope, highlighting significant opportunities for further exploration in this area. Given the importance of epoxides, there remains a substantial unresolved need to design novel, straightforward, and widely applicable epoxidation methods. Incorporating these important motifs into complex molecules *via* such strategies requires further investigation.

## 6 Synthesis of other three-membered rings by photo/electrochemical approaches

In addition to the syntheses of cyclopropanes, aziridines, and epoxides, methods for synthesizing cyclopropenes, vinyloxaziridines, and azirines have also been reported.^91,92,95,96^ Although some photo/electrochemical methods have been developed, there remains a demand for alternative pathways to construct these compounds, compared to cyclopropanes or aziridines.

**Scheme 14:**
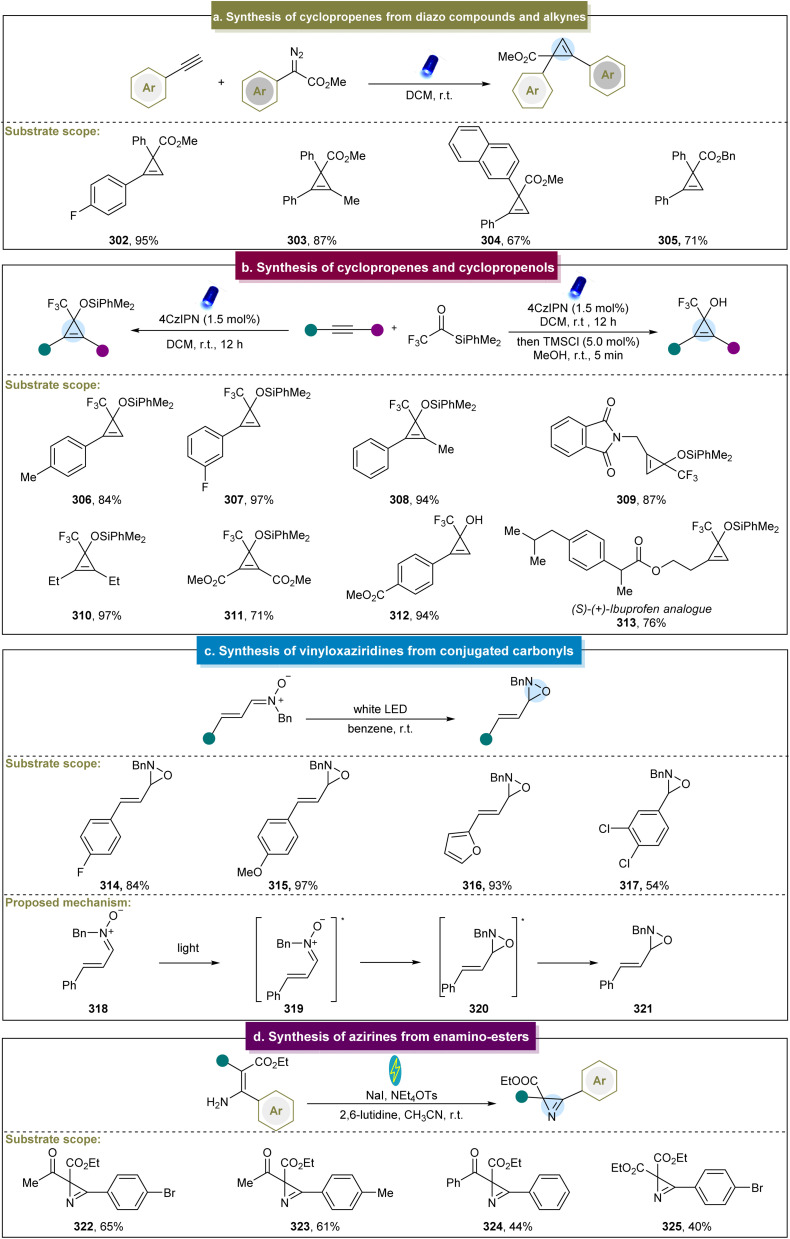
(a) Cyclopropenation of diazo compounds with alkynes. (b) Synthesis of cyclopropenes and cyclopropenols. (c) Synthesis of vinyloxaziridines from conjugated carbonyls. (d) Synthesis of azirines from enamino-esters.

In 2018, Königs *et al.* reported visible-light-induced cyclopropenation between diazo esters and aryl alkynes.^91^ Various electron-donating and electron-withdrawing arylacetylenes efficiently produced the desired cyclopropenes in high yields, such as 302. Moreover, 1,2-diaryl alkynes or alkyl-aryl-substituted alkynes also yielded the target products (Scheme 14a, 303–305). Notably, this reaction was conducted rapidly in continuous-flow devices, which significantly enhanced the reaction yield. This approach offered several advantages, including the absence of metal catalysts, no need for air exclusion, and straightforward operation. However, the reaction exhibited limited reactivity towards heterocyclic aryl acetylenes. Subsequently, they reported photochemical carbene transfer reactions using diazoesters as the carbene precursors, which enabled cyclopropenation with unprotected propargyl alcohols.^173^ Unfortunately, the diazoesters reacted effectively only with the propargyl alcohols bearing electron-donating groups, restricting the scope of the reaction.

In 2021, researchers continued their investigations in this field and reported the photochemical cyclopropenation of aryl/aryl diazoalkanes with alkynes.^174^ Depending on the electronic nature of different substituents, selective generation of different products was achieved. Detailed calculations and experimental studies have indicated that the introduction of electron-donating and electron-withdrawing groups can significantly affect the singlet–triplet energy level splitting of diaryl carbene intermediates and the activation energy of consecutive carbene transfer reactions. This strategy can overcome the classical limitations of diaryl carbene reactions, enabling the aryl/aryl diazoalkanes to participate in photochemical carbene transfer reactions controlled by the singlet and triplet spin states. In addition to the diazo pathway, Shen *et al.* first reported a visible light-induced [2 + 1] cycloaddition reaction for the synthesis of cyclopropenes and cyclopropenols.^92^ The key intermediate in this reaction, α-CF_3_ siloxycarbene, served as the donor–acceptor-type carbene. Various terminal and internal aryl alkynes were compatible with this reaction system (Scheme 14b, 306–312), demonstrating good adaptability even in complex biologically active molecules such as (*S*)–(+)-ibuprofen derivative 313. Moreover, this strategy efficiently transformed the obtained cyclopropenes into cyclopropenols in high yields in a one-pot manner.

In addition to the synthesis of cyclopropenes, Moura-Letts *et al.* reported a method for synthesizing vinyloxaziridines *via* visible-light-induced energy transfer.^95^ The main mechanism involved the condensation reaction between the conjugated carbonyls and hydroxylamines (Scheme 14c). The generated vinyl nitrones underwent photocatalytic isomerization to yield the final products. This reaction utilized simple vinyl nitrones as the starting materials and achieved high yields and stereoselectivity in the formation of vinyloxaziridines (314–317). However, the substrate scope of this method was limited. Furthermore, Hilt *et al.* introduced an electrochemical method for the intramolecular synthesis of azirines from enamino esters (Scheme 14d, 322–325), and the divided cell was equipped with graphite, and platinum was used as the cathode and electrolyzed at a constant current.^96^ The success of this strategy relied on the generation of intermediate *N*-indoamines, offering a novel approach for synthesizing azirines. Furthermore, these azirines can serve as the building blocks to efficiently produce valuable 4-carboxy-oxazoles in high yields, thereby demonstrating the practicality of this method.

Overall, the synthesis of cyclopropenes, vinyloxaziridines, and azirines using photo/chemical methods remained underexplored. Current approaches for the cyclopropene synthesis typically involved the generation of specific carbene intermediates to react with alkynes, yielding the desired products. However, these carbene precursors often required electron-withdrawing groups, highlighting the need for broader exploration of more versatile carbene sources. Additionally, electrocatalytic methods for the synthesis of cyclopropene have not yet been reported. Given the potential pharmaceutical relevance of these structures, further efforts are required to enhance and diversify their synthetic pathways.

## 7 Conclusions

In conclusion, this review comprehensively outlines the synthesis of three-membered rings using photo/electrochemical approaches. These updates demonstrated the importance of photo/electrochemical methods as viable alternatives for synthesizing these rings, eliminating the need for traditional thermal- and transition-metal-catalyzed conditions. Furthermore, these sustainable methods have diversified the repertoire of three-membered rings, thereby expanding the chemical space relevant to drug discovery. Photo/electrochemical strategies not only enhanced the synthetic efficiency and structural diversity, but also promoted the high atom economy in obtaining these molecules. However, several opportunities and challenges have persisted in this field. For instance, a few studies have demonstrated practical applications, such as the gram-scale synthesis, late-stage modifications, and total synthesis of pharmaceuticals or natural products. Furthermore, tailored substrates were crucial for effective activation. However, these innovative methods have not been widely adopted in the industry. Despite the advancements in structural diversity, synthesizing highly congested three-membered rings has remained challenging owing to the steric hindrance, necessitating continued efforts to overcome this limitation. The enantioselective synthesis of three-membered rings using photo/electrochemical strategies remains a relatively unexplored area. Given the significance of chiral molecules in drug discovery, this challenge is expected to attract increased attention in the future. The advances in synthetic biology and enzyme catalysis have suggested that the enzyme-photo-coupled catalytic systems can facilitate the efficient and rapid synthesis of chiral cyclopropanes. Overall, this review could provide insights into the recent advancements in three-membered ring synthesis *via* photo/electrochemical methods and inspire further progress in this field in the near future.

## Data availability

All data are available from the authors on request.

## Author contributions

Y. Z. and S. D. conceived the project. Y. Z., N. Z. and H. X. conducted the literature survey. H. X., N. Z. and Q. L. wrote the manuscript. Y. Z. and S. D. guided and reviewed the manuscript. All authors have given approval to the final version of the manuscript.

## Conflicts of interest

There are no conflicts to declare.

## References

[cit1] Carson C. A., Kerr M. A. (2009). Chem. Soc. Rev..

[cit2] de Meijere A., Kozhushkov S. I., Schill H. (2006). Chem. Rev..

[cit3] Gagnon A., Duplessis M., Fader L. (2010). Org. Prep. Proced. Int..

[cit4] Gomes A. R., Varela C. L., Tavares-da-Silva E. J., Roleira F. M. (2020). Eur. J. Med. Chem..

[cit5] Singh G. S. (2016). Mini-Rev. Med. Chem..

[cit6] Dahlman D. L., Kadaba P. K. (1988). Pestic. Sci..

[cit7] Xiao P., Mori T., Kamei I., Kondo R. (2011). FEMS Microbiol. Lett..

[cit8] Luo M., Zhang X. H., Darensbourg D. J. (2016). Acc. Chem. Res..

[cit9] Childers M. I., Longo J. M., Van Zee N. J., LaPointe A. M., Coates G. W. (2014). Chem. Rev..

[cit10] McGrath N. A., Brichacek M., Njardarson J. T. (2010). J. Chem. Educ..

[cit11] Littleson M. M., Baker C. M., Dalençon A. J., Frye E. C., Jamieson C., Kennedy A. R., Ling K. B., McLachlan M. M., Montgomery M. G., Russell C. J., Watson A. J. (2018). Nat. Commun..

[cit12] Yamada K., Ojika M., Kigoshi H. (2007). Nat. Prod. Rep..

[cit13] Hernandez K. E., Renata H., Lewis R. D., Kan S. J., Zhang C., Forte J., Rozzell D., McIntosh J. A., Arnold F. H. (2016). ACS Catal..

[cit14] Chen D. Y. K., Pouwer R. H., Richard J. A. (2012). Chem. Soc. Rev..

[cit15] Thibodeaux C. J., Chang W., Liu H. (2012). Chem. Rev..

[cit16] Ma S., Mandalapu D., Wang S., Zhang Q. (2022). Nat. Prod. Rep..

[cit17] Rajski S. R., Williams R. M. (1998). Chem. Rev..

[cit18] Armstrong R. W., Salvati M. E., Nguyen M. (1992). J. Am. Chem. Soc..

[cit19] Tsuchida T., Sawa R., Takahashi Y., Iinuma H., Sawa T., Naganawa H., Takeuchi T. (1995). J. Antibiot..

[cit20] Salaiin J., Bairtr M. (1995). Curr. Med. Chem..

[cit21] Ebner C., Carreira E. M. (2017). Chem. Rev..

[cit22] Brandi A., Cicchi S., Cordero F. M., Goti A. (2003). Chem. Rev..

[cit23] Reissig H. U., Zimmer R. (2003). Chem. Rev..

[cit24] Nagarajan M., Morrell A., Ioanoviciu A., Antony S., Kohlhagen G., Agama K., Hollingshead M., Pommier Y., Cushman M. (2006). J. Med. Chem..

[cit25] Osborn H. M. I., Sweeney J. (1997). Tetrahedron: Asymmetry.

[cit26] Huang L., Wulff W. D. (2011). J. Am. Chem. Soc..

[cit27] Simmons H. E., Smith R. D. (1958). J. Am. Chem. Soc..

[cit28] Wessjohann L. A., Brandt W., Thiemann T. (2003). Chem. Rev..

[cit29] Simmons H. E., Cairns T. L., Vladuchick S. A., Hoiness C. M. (2004). Org. React..

[cit30] Davies H. M., Walji A. M. (2005). Org. lett..

[cit31] Crawford R. J., Ohno M. (1974). Can. J. Chem..

[cit32] Kulinkovich O. G. (2003). Chem. Rev..

[cit33] Herraiz A. G., Suero M. G. (2019). Synthesis.

[cit34] Burke S. D., Grieco P. A. (2004). Org. React..

[cit35] Li X., Chen N., Xu J. (2010). Synthesis.

[cit36] Callebaut G., Meiresonne T., De Kimpe N., Mangelinckx S. (2014). Chem. Rev..

[cit37] Degennaro L., Trinchera P., Luisi R. (2014). Chem. Rev..

[cit38] Dequirez G., Pons V., Dauban P. (2012). Angew. Chem., Int. Ed..

[cit39] Jiang H., Lang K., Lu H., Wojtas L., Zhang X. P. (2017). J. Am. Chem. Soc..

[cit40] Thakur V. V., Sudalai A. (2003). Tetrahedron Lett..

[cit41] Cheng Q. Q., Zhou Z., Jiang H., Siitonen J. H., Ess D. H., Zhang X., Kürti L. (2020). Nat. Catal..

[cit42] Bieszczad B., Chen X., Zard S. Z. (2022). Org. Lett..

[cit43] Mello R., Alcalde-Aragonés A., González Núñez M. E., Asensio G. (2012). J. Org. Chem..

[cit44] Nam D. G., Shim S. Y., Jeong H. M., Ryu D. H. (2021). Angew. Chem., Int. Ed..

[cit45] Johnson A. W., LaCount R. B. (1961). J. Am. Chem. Soc..

[cit46] Zhu R., Buchwald S. L. (2012). J. Am. Chem. Soc..

[cit47] He Q., Pu M. P., Jiang Z., Wang H., Feng X., Liu X. (2023). J. Am. Chem. Soc..

[cit48] Binger P., Buch H. M. (1987). Top. Curr. Chem..

[cit49] Carter F. L., Frampton V. L. (1964). Chem. Rev..

[cit50] Nakamura M., Isobe H., Nakamura E. (2003). Chem. Rev..

[cit51] Fox J. M., Yan N. (2005). Curr. Org. Chem..

[cit52] Marek I., Simaan S., Masarwa A. (2007). Angew. Chem., Int. Ed..

[cit53] Aubé J. (1997). Chem. Soc. Rev..

[cit54] Allen C. P., Benkovics T., Turek A. K., Yoon T. P. (2009). J. Am. Chem. Soc..

[cit55] Michaelis D. J., Shaffer C. J., Yoon T. P. (2007). J. Am. Chem. Soc..

[cit56] Patonay T., Jeko J., Juhμsz-Tóth É. (2008). Eur. J. Org Chem..

[cit57] Zhao M. N., Zhang W., Wang X. C., Zhang Y., Yang D. S., Guan Z. H. (2018). Org. Biomol. Chem..

[cit58] Okamoto K., Nanya A., Eguchi A., Ohe K. (2018). Angew. Chem., Int. Ed..

[cit59] Chen D. Y. K., Pouwer R. H., Richard J. A. (2012). Chem. Soc. Rev..

[cit60] Narayanam J. M. R., Stephenson C. R. (2011). J. Chem. Soc. Rev..

[cit61] Narayanam J. M. R., Tucker J. W., Stephenson C. R. J. (2009). J. Am. Chem. Soc..

[cit62] Cauwenbergh R., Das S. (2022). Synlett.

[cit63] Jaro V., Das S. (2022). Synthesis.

[cit64] Feng T., Wang S., Liu Y., Liu S., Qiu Y. (2022). Angew. Chem., Int. Ed..

[cit65] Guillemard L., Kaplaneris N., Ackermann L., Johansson M. J. (2021). Nat. Rev. Chem.

[cit66] Tay N. E., Lehnherr D., Rovis T. (2021). Chem. Rev..

[cit67] Liu J. Q., Shatskiy A., Kärkäs M. D. (2020). Science.

[cit68] Del Hoyo A. M., Herraiz A. G., Suero M. G. (2017). Angew. Chem., Int. Ed..

[cit69] Shu C., Mega R. S., Andreassen B. J., Noble A., Aggarwal V. K. (2018). Angew. Chem., Int. Ed..

[cit70] Phelan J. P., Lang S. B., Compton J. S., Kelly C. B., Dykstra R., Gutierrez O., Molander G. A. (2018). J. Am. Chem. Soc..

[cit71] Chidley T., Jameel I., Rizwan S., Peixoto P. A., Pouységu L., Quideau S., Murphy G. K. (2019). Angew. Chem., Int. Ed..

[cit72] Poudel D. P., Pokhrel A., Tak R. K., Shankar M., Giri R. (2023). Science.

[cit73] Wang Z., Herraiz A. G., Del Hoyo A. M., Suero M. G. (2018). Nature.

[cit74] Zhang Y., Zhou G., Gong X., Guo Z., Qi X., Shen X. (2022). Angew. Chem., Int. Ed..

[cit75] Xia D., Wu R., Wang J., Han X., Li Y., Li Q., Luan X., Hong X., Zhang Y., Zhang W. D. (2023). ACS Catal..

[cit76] Sarabia F. J., Ferreira E. M. (2017). Org. Lett..

[cit77] Xu B., Troian-Gautier L., Dykstra R., Martin R. T., Gutierrez O., Tambar U. K. (2020). J. Am. Chem. Soc..

[cit78] Kim M. J., Wang D. J., Targos K., Garcia U. A., Harris A. F., Guzei I. A., Wickens Z. K. (2023). Angew. Chem., Int. Ed..

[cit79] Scholz S. O., Farney E. P., Kim S., Bates D. M., Yoon T. P. (2016). Angew. Chem., Int. Ed..

[cit80] Guo Y., Pei C., Jana S., Koenigs R. M. (2021). ACS Catal..

[cit81] Chen J., Yan W. Q., Lam C. M., Zeng C. C., Hu L. M., Little R. D. (2015). Org. Lett..

[cit82] Liu F., Dai J., Cheng X. (2021). Chin. J. Org. Chem..

[cit83] Liu Y., Dong X., Deng G. L., Zhou L. (2016). Sci. China. Chem..

[cit84] Holst D. E., Wang D. J., Kim M. J., Guzei I. A., Wickens Z. K. (2021). Nature.

[cit85] Liu X. X., Jia J., Wang Z., Zhang Y. T., Chen J., Yang K., He C. Y., Zhao L. (2020). Adv. Synth. Catal..

[cit86] Chand S., Sharma A. K., Pandey A. K., Singh K. N. (2022). Org. Lett..

[cit87] Kim E., Choi S., Kim H., Cho E. J. (2013). Chem.–Eur. J..

[cit88] Ines M., Mendonca A. J., Esteves A. P., Mendonca D. I., Medeiros M. J. (2009). C. R. Chim..

[cit89] Jin K., Maalouf J. H., Lazouski N., Corbin N., Yang D., Manthiram K. (2019). J. Am. Chem. Soc..

[cit90] Leow W. R., Lum Y., Ozden A., Wang Y., Nam D.-H., Chen B., Wicks J., Zhuang T. T., Li F., Sinton D., Sargen E. H. (2020). Science.

[cit91] Hommelsheim R., Guo Y., Yang Z., Empel C., Koenigs R. M. (2019). Angew. Chem., Int. Ed..

[cit92] Zhou G., Shen X. (2022). Angew. Chem., Int. Ed..

[cit93] Li J., Lear M. J., Hayashi Y. (2021). Chem.–Eur. J..

[cit94] Guo Y., Empel C., Pei C., Fang H., Jana S., Koenigs R. M. (2022). Chem Catal..

[cit95] Austin B. E., Palner R. P., Tobias E. M., Madiu R., Doran E. L., Doran J.
M., Doran J. M., Howard A. M., Stroud J. L., Rossi M. E., Moskovitz D. A., Rivera D. A., Mullen M. D., Zinsky A. H., Rosario R. A., Moura-Letts G. (2024). Synlett.

[cit96] Babaoglu E., Hilt G. (2020). Chem.–Eur. J..

[cit97] Sengmany S., Léonel E., Paugam J. P., Nédélec J. Y. (2002). Synthesis.

[cit98] Ribeiro R. T., De Mattos I. L., Sengmany S., Barhdadi R., Léonel E., Cachet-Vivier C., Navarro M. (2011). Electrochim. Acta.

[cit99] Zhang Y., Qian R., Zheng X., Zeng Y., Sun J., Chen Y., Ding A., Guo H. (2015). Chem. Commun..

[cit100] del Hoyo A. M., Suero M. G. (2017). Eur. J. Org Chem..

[cit101] Li P., Zhao J., Shi L., Wang J., Shi X., Li F. (2018). Nat. Commun..

[cit102] Ohtani T., Tsuchiya Y., Uraguchi D., Ooi T. (2019). Org. Chem. Front..

[cit103] Herraiz A. G., Suero M. G. (2019). Chem. Sci..

[cit104] Ide K., Furuta M., Tokuyama H. (2021). Org. Biomol. Chem..

[cit105] Jana S. K., Maiti M., Dey P., Maji B. (2022). Org. Lett..

[cit106] Luo S. S., Shen H., Li S. J., Cao T., Luo Y. P., Zhang S., Zhou T., Liu X. W. (2022). Org. Chem. Front..

[cit107] Fischer D. M., Lindner H., Amberg W. M., Carreira E. M. (2023). J. Am. Chem. Soc..

[cit108] Ji C. L., Han J., Li T., Zhao C. G., Zhu C., Xie J. (2022). Nat. Catal..

[cit109] Thai-Savard L., Sayes M., Perreault-Dufour J., Hong G., Wells L. A., Kozlowski M. C., Charette A. B. (2023). J. Org. Chem..

[cit110] Bosveli A., Griboura N., Kampouropoulos I., Kalaitzakis D., Montagnon T., Vassilikogiannakis G. (2023). Chem.–Eur. J..

[cit111] Biswas S., Chandu P., Garai S., Sureshkumar D. (2023). Org. Lett..

[cit112] Guo T., Zhang L., Liu X., Fang Y., Jin X., Yang Y., Li Y., Chen B., Ouyang M. (2018). Adv. Synth. Catal..

[cit113] Milligan J. A., Phelan J. P., Polites V. C., Kelly C. B., Molander G. A. (2018). Org. Lett..

[cit114] Luo W., Yang Y., Fang Y., Zhang X., Jin X., Zhao G., Zhang L., Li Y., Zhou W., Xia T., Chen B. (2019). Adv. Synth. Catal..

[cit115] Luo W., Fang Y., Zhang L., Xu T., Liu Y., Li Y., Jin X., Bao J., Wu X., Zhang Z. (2020). Eur. J. Org Chem..

[cit116] Liu Y., Luo W., Wu J., Fang Y., Li Y., Jin X., Zhang L., Zhang Z., Xu F., Du C. (2020). Org. Chem. Front..

[cit117] Nakamura R., Sumida Y., Ohmiya H. (2022). Bull. Chem. Soc. Jpn..

[cit118] Huang X. L., Cheng Y. Z., You S. L. (2022). Org. Chem. Front..

[cit119] Zhang L., Shi J., Fang Y. (2023). Synthesis.

[cit120] Huai L., Zhang L., Wang Z., Wu H., Fang Y. (2023). Org. Chem. Front..

[cit121] Hu J., Tang M., Wang J., Wu Z., Friedrich A., Marder T. B. (2023). Angew. Chem., Int. Ed..

[cit122] Jie L. H., Guo B., Song J., Xu H. C. (2022). J. Am. Chem. Soc..

[cit123] Cai B. G., Empel C., Jana S., Xuan J., Koenigs R. M. (2023). ACS Catal..

[cit124] Teye-Kau J. H. G., Ayodele M. J., Pitre S. (2023). Angew. Chem., Int. Ed..

[cit125] Zhang X., Du C., Zhang H., Li X. C., Wang Y. L., Niu J. L., Song M. P. (2019). Synthesis.

[cit126] Jurberg I. D., Davies H. M. (2018). Chem. Sci..

[cit127] Guo Y., Nguyen T. V., Koenigs R. M. (2019). Org. Lett..

[cit128] Jana S., Li F., Empel C., Verspeek D., Aseeva P., Koenigs R. M. (2020). Chem.–Eur. J..

[cit129] Chauhan J., Ravva M. K., Gremaud L., Sen S. (2020). Org. Lett..

[cit130] Guo Y., Empel C., Pei C., Atodiresei I., Fallon T., Koenigs R. M. (2020). Org. Lett..

[cit131] Empel C., Koenigs R. M. (2020). J. Flow Chem..

[cit132] Klöpfer V., Eckl R., Floß J., Roth P. M., Reiser O., Barham J. P. (2021). Green Chem..

[cit133] Qiu H., Wen L., Lv J. (2022). Synthesis.

[cit134] Chen Z. L., Empel C., Wang K., Wu P. P., Cai B. G., Li L., Xuan J. (2022). Org. Lett..

[cit135] Xu Y., Lv G., Yan K., He H., Li J., Luo Y., Lai R., Li H., Wu Y. (2020). Chem.–Asian J..

[cit136] George V., König B. (2023). Chem. Commun..

[cit137] He F., Sun Z., Li C., Jiang Z., Miao H., Li Q., Wu C. (2023). Org. Biomol. Chem..

[cit138] Yamaguchi E., Inagawa H., Itoh A. (2022). Photochem. Photobiol. Sci..

[cit139] Bunyamin A., Hua C., Polyzos A., Priebbenow D. L. (2022). Chem. Sci..

[cit140] Chen S., Zhang Y., Liu S., Shen X. (2023). Sci. China: Chem..

[cit141] Orłowska K., Santiago J. V., Krajewski P., Kisiel K., Deperasińska I., Zawada K., Chaładaj W., Gryko D. (2023). ACS Catal..

[cit142] Deng Y., Zhang J., Bankhead B., Markham J. P., Zeller M. (2021). Chem. Commun..

[cit143] Richardson A. D., Vogel T. R., Traficante E. F., Glover K. J., Schindler C. S. (2022). Angew. Chem., Int. Ed..

[cit144] Ma J., Chen S., Bellotti P., Wagener T., Daniliuc C., Houk K. N., Glorius F. (2022). Nat. Catal..

[cit145] Vereshchagin A. N., Dorofeeva E. O., Elinson M. N., Egorov M. P. (2019). Arkivoc.

[cit146] Matsumoto K., Hayashi Y., Hamasaki K., Matsuse M., Suzuki H., Nishiwaki K., Kawashita N. (2022). Beilstein J. Org. Chem..

[cit147] Cantin T., Charette A. B., Poisson T., Jubault P. (2023). Synthesis.

[cit148] Zhang Y., Dong X., Wu Y., L G., Lu H. (2018). Org. Lett..

[cit149] Xing Q., Jiang D., Zhang J., Guan L., Li T., Zhao Y., Di M., Chen H., Che C., Zhu Z. (2022). Commun. Chem..

[cit150] Shikhar Srivastava O., Anand V., Rastogi N. (2023). Asian J. Org. Chem..

[cit151] Cheng X., Cai B. G., Mao H., Lu J., Li L., Wang K., Xuan J. (2021). Org. Lett..

[cit152] Siu T., Yudin A. K. (2002). J. Am. Chem. Soc..

[cit153] Matsuzawa K., Nagasawa Y., Yamaguchi E., Tada N., Itoh A. (2016). Synthesis.

[cit154] Yu W. L., Chen J. Q., Wei Y. L., Wang Z. Y., Xu P. F. (2018). Chem. Commun..

[cit155] Zeng D., Gu L., Zhang L., Li G., He Y. (2021). Tetrahedron Lett..

[cit156] Vanhoof J. R., De Smedt P. J., Krasniqi B., Ameloot R., Sakellariou D., De Vos D. E. (2021). ACS Sustainable Chem. Eng..

[cit157] Li J., Huang W., Chen J., He L., Cheng X., Li G. (2018). Angew. Chem., Int. Ed..

[cit158] Ošeka M., Laudadio G., van Leest N. P., Dyga M., Bartolomeu A. D. A., Gooßen L. J., de Bruin B., de Oliveira K. T., Noël T. (2021). Chem.

[cit159] Wang S., Wang P., Li S. J., Chen Y. H., Sun Z. J., Lei A. (2023). Nati. Sci. Rev..

[cit160] Mohsenzadeh A., Zamani A., Taherzadeh M. J. (2017). ChemBioEng Rev..

[cit161] Lucky C., Wang T., Schreier M. (2022). ACS Energy Lett..

[cit162] Tang D., Dang K., Wang J., Chen C., Zhao J., Zhang Y. (2023). Sci. China: Chem..

[cit163] Kumar R., Banerjee N., Kumar P., Banerjee P. (2023). Chem.–Eur. J..

[cit164] Tada N., Okubo H., Miura T., Itoh A. (2009). Synlett.

[cit165] Tanaka H., Nishikawa H., Uchida T., Katsuki T. (2010). J. Am. Chem. Soc..

[cit166] Wu Y., Zhou G., Meng Q., Tang X., Liu G., Yin H., Zhao J., Yang F., Yu Z., Luo Y. (2018). J. Org. Chem..

[cit167] Luo K., Yu X., Chen P., He K., Lin J., Jin Y. (2020). Tetrahedron Lett..

[cit168] Peault L., Rochelle S., Planchat A., Nun P., Le Grognec E., Coeffard V. (2023). Adv. Synth. Catal..

[cit169] Zhang Y., Yang X., Tang H., Liang D., Wu J., Huang D. (2020). Green Chem..

[cit170] Shen D., Ren T., Luo Z., Sun F., Han Y., Chen K., Zhang X., Zhou M., Gong P., Chao M. (2023). Org. Biomol. Chem..

[cit171] Omura R., Fujiya A., Yamaguchi E., Tada N., Miura T., Itoh A. (2016). Synthesis.

[cit172] Tang H., Vanhoof J. R., De Vos D. (2022). Green Chem..

[cit173] He F., Koenigs R. M. (2019). Chem. Commun..

[cit174] Jana S., Pei C., Empel C., Koenigs R. M. (2021). Angew. Chem., Int. Ed..

